# Serum Lipid Reference Intervals of High-Density, Low-Density and Non-High-Density Lipoprotein Cholesterols and Their Association with Atherosclerosis and Other Factors in Psittaciformes

**DOI:** 10.3390/ani15172493

**Published:** 2025-08-25

**Authors:** Matthias Janeczek, Rüdiger Korbel, Friedrich Janeczek, Helen Alber, Helmut Küchenhoff, Monika Rinder

**Affiliations:** 1 Clinic for Birds, Small Mammals, Reptiles and Ornamental Fish, Centre for Clinical Veterinary Medicine, Ludwig-Maximilians-Universität München, 85764 Oberschleißheim, Germany; korbel@lmu.de (R.K.); monika.rinder@lmu.de (M.R.); 2 Veterinary Practice for Parrots, 82166 Gräfelfing, Germany; info@janeczek.de; 3 Statistical Consulting Unit StaBLab, Department of Statistics, Ludwig-Maximilians-Universität München, 80539 Munich, Germany; helen.alber@stat.uni-muenchen.de (H.A.); kuechenhoff@stat.uni-muenchen.de (H.K.)

**Keywords:** parrots, lipoproteins, HDL, LDL, non-HDL, cholesterol, atherosclerosis

## Abstract

Atherosclerosis is highly prevalent among captive psittacine populations and is a frequent cause of veterinary consultations. Ante-mortem diagnosis remains challenging, but the serum lipoprotein analysis has been suggested as a useful tool for identifying associated risk factors and improving understanding of its pathogenesis. Unlike in humans, the relationship between lipoproteins and atherosclerosis in parrots has not been clearly established. This retrospective cohort study analyzed *n* = 1199 blood samples from 692 parrots across 14 genera to establish reference intervals for high-density lipoprotein cholesterol (HDL-C), low-density lipoprotein cholesterol (LDL-C) and non-high-density lipoprotein cholesterol (non-HDL-C) following ASVCP guidelines. Lipoprotein levels were evaluated in relation to factors such as genus, age, sex, diet, reproductive status, body condition score, and atherosclerosis prevalence (diagnosed by endoscopy and/or necropsy). The results demonstrated genus-specific differences and significant associations between LDL-C and atherosclerosis, with non-HDL-C showing a similar, less pronounced, trend. Higher LDL-C values were measured in the presence of moderate-severe atherosclerosis. Birds on seed diets had higher lipoprotein levels and were more likely to be diagnosed with atherosclerosis in comparison to birds fed a pelleted or extruded diet. The role of HDL-C remained less conclusively defined. The results of this study provide a foundational framework for the future use of lipoprotein analysis in parrot medicine, offering novel insights into the management of cardiovascular health in pet parrots.

## Simple Summary

Parrots are popular pets known for their long lifespans. They often suffer from a disease called atherosclerosis, where fatty lesions build up and clog their blood vessels. This disease is particularly hard to diagnose in its early stages. In humans, cholesterol is known as a risk factor for the development atherosclerosis, which is why this study aimed to better understand cholesterol levels in parrots and if they relate to this disease. Blood samples from 1199 parrots of different species were used to determine concentrations of various types of cholesterol. It was found that cholesterol levels vary between parrot species and are influenced by factors like diet, age, body condition, and whether the bird is breeding. Birds on healthy, balanced diets had lower levels of LDL-C, a certain type of cholesterol. Higher levels of the same cholesterol type were additionally linked to more severe atherosclerosis, though these levels alone could not predict the disease. This study provides useful reference ranges for cholesterols in parrots which could lead to better prevention and treatment of cardiovascular disease, improving the health and lifespan of pet parrots.

## 1. Introduction

Parrots (Psittaciformes) have been kept as companion animals for centuries with an increase in popularity in the late 20th century [1]. With time, we have gained a better understanding of their biology, husbandry and disease patterns [2]. The parrot population in the Western Hemisphere, which originated from large-scale imports that are prohibited today [3], grew older, and with rising age, avian veterinarians were increasingly confronted with the importance of managing poor husbandry and the emergence of geriatric diseases.

Parrots are a zoological order of long-living birds with the genetic potential to reach ages documented up to 88 years [4], depending on genus, species, as well as proper diet and husbandry. With progressing age of parrots, avian veterinarians are confronted with similar geriatric diseases as in humans, as older birds often develop arthrosis, cataracts, and cardiovascular disease [5]. Atherosclerosis, in particular, has become the cardiovascular lesion with the highest prevalence in captive parrot populations with documented prevalences up to 91.8%, and is a commonly reported secondary finding in post-mortem examinations, even if histologically detectable lesions may not yet be clinically relevant [6,7,8,9]. Atherosclerosis is generally characterized as a chronic inflammatory disease of the arterial wall [10]. The accumulation of foam cells, lipids, calcium, and cellular debris leads to atheromatous plaques that narrow the arterial lumen. In parrots, the lesions themselves often remain in a subclinical state and are difficult to diagnose in their initial stage [7,11]. The ante-mortem suspicion of lesions is commonly based on the emergence of actual clinical signs, mostly attributed to flow-limited stenosis of advanced lesions, such as exercise intolerance, weakness, respiratory signs, congestive heart failure, neurologic signs, or even sudden death [6,7,12]. However, even in that stage, diagnosis is oftentimes difficult, as diagnostics frequently have low sensitivity and specificity, are high in cost, or invasive [7,11,13,14,15,16,17]. Especially in the early stages without obvious calcifications, it can be difficult to detect in the small arterial lumen of birds. Diagnostic measures include radiography, computed tomography, echocardiography, fluoroscopic angiography, blood pressure measurements, endoscopy, or nuclear imaging [14,18]. A novel technique, micro-positron emission tomography, showed promising results for the diagnosis of atherosclerosis [18], but is currently not yet available for the use in daily practice. In general, to this day, a standardized diagnostic protocol with high sensitivity and specificity remains to be established.

In humans in the Western world, atherosclerosis and subsequent ischemic heart disease is the main cause of death, followed by strokes [19,20]. Given its high clinical relevance, atherosclerosis has been the focus of extensive research, in which animal models, both mammalian and avian, played a key role [21], leading to a well-established understanding of its pathogenesis, clinical course, and therapeutic interventions. Influenceable predisposing factors were identified and found to include a sedentary lifestyle, obesity, diabetes mellitus, smoking, and many others, with hypercholesterolemia, high low-density lipoprotein (LDL) and low high-density lipoprotein (HDL) being key factors in the development of atherosclerosis [22,23], raising the question about their role in birds, who are also highly susceptible to atherosclerotic cardiovascular disease. Lipid-lowering drugs, primarily statins, are a main focus of therapy in humans with atorvastatin and rosuvastatin most commonly used in the treatment of dyslipidemias and atherosclerosis [24]. First studies about their pharmacokinetics, dynamics, and potential therapeutic value in psittacines have been published [25,26,27,28].

Lipoproteins are molecular structures that consist of proteins and lipids, mainly cholesterol, triglycerides, and phospholipids, that are formed in the liver of mammals, but also of birds [29]. It is generally known that they play a crucial role in transporting lipids through the bloodstream to the peripheral tissues, where the lipids are used for energy utilization, lipid deposition, steroid hormone production, and bile acid formation. Very-low-density lipoprotein (VLDL) is synthesized in the liver and transports triglycerides. As VLDL loses triglycerides, it converts into intermediate-density lipoprotein (IDL), which can either be taken up by the liver or further processed into LDL. LDL transports cholesterol from the liver to peripheral tissues and consists of high amounts of cholesterol and few triglycerides. In contrast, HDL carries more protein, is of a higher density than LDL, and removes excess cholesterol from the bloodstream and tissues, transporting it back to the liver for excretion or recycling. Maintaining a balance between the various lipoproteins is essential for overall health. Dysregulation of lipoprotein metabolism may lead to various health problems, including atherosclerosis, heart disease, and metabolic disorders [10,30]. The opportunity to evaluate and properly understand additional lipoprotein data in HDL and LDL might further aid the diagnosis of dyslipidemias and subsequent diseases like atherosclerosis, gonadal diseases, endocrine disease, and nutritional diseases in parrots.

HDL and LDL are more correctly referred to as HDL-Cholesterol (HDL-C) and LDL-Cholesterol (LDL-C) in laboratory medicine, because in most tests not the concentration of HDL and LDL, but their cholesterol concentration in the serum, is actually being measured. The nomenclature of HDL and LDL is appropriate, if the particle numbers of HDL and LDL are measured, which is possible, but less frequently performed in avian medicine [31,32]. There are different methods available for the determination of these values. High performance liquid chromatography (HPLC) is considered the gold standard; however, while it is very accurate, it is less available and linked to high costs [31,32]. Another option is the reasonably priced, frequently available enzymatic and photometric determination via benchtop biochemistry analyzer. Measurement of HDL-C has been proven to be very accurate in this method, while LDL-C provides less precise data compared to HPLC, which is why it has been proposed to instead calculate non-high-density lipoprotein cholesterol (non-HDL-C) [31]. Non-HDL-C encompasses various lipoproteins, including LDL-C, VLDL-C, IDL-C, and portomicrons [31]. The Friedewald formula is an indirect method to calculate LDL-C values in humans, but has been shown to be inaccurate for parrots, especially in lipemic samples [31,32]. Another method, that may potentially be useful in psittacines and shows good accuracy in human medicine, is lipoprotein electrophoresis [33]. While testing blood chemistry values in clinical situations in parrot medicine, the lipid panel, typically consisting of total cholesterol (TC), triglycerides, HDL-C, and LDL-C, is not commonly used. Though TC and triglycerides are sometimes evaluated, the literature lacks information on the diagnostic value of HDL-C, LDL-C, and non-HDL-C, and there are few data on reference ranges for many different genera of parrots [34,35,36].

The goals of this study were as follows: First, to establish reference intervals for enzymatic and colorimetric analyses for HDL-C, LDL-C, and non-HDL-C serum concentrations in different parrot genera. Second, serum HDL-C, LDL-C, and non-HDL-C concentrations were to be evaluated further, to understand how the values change depending on different internal and external factors, and how LDL-C and non-HDL-C compare to each other. More specifically, how the values change depending on age, sex, diet, body condition score (BCS), reproductive activity, and actual prevalence of atherosclerosis. Third, birds with diagnosed atherosclerosis were observed based on the same factors, now including their individual HDL-C, LDL-C, and non-HDL-C parameters, to evaluate the prevalence and risk factors associated with atherosclerosis in psittacine birds.

The hypotheses were that different parrot genera would have different reference intervals, and values would be higher for birds with unbalanced diets (which do not fully meet the bird’s needs), reproductive activity, elevated body conditioning scores, and atherosclerotic disease, as well as higher values with rising age and in female sex. In addition, we expected birds would be more likely to develop atherosclerosis the higher their levels of LDL-C and non-HDL-C were, and the lower HDL-C was. The same was hypothesized to be valid for different genera, rising age, female sex, elevated body conditioning score, unbalanced diet, and breeding birds.

## 2. Materials and Methods

### 2.1. Study Animals and Sample Collection

A total of *n* = 1196 HDL-C and 1190 LDL-C serum values were collected from *n* = 1199 blood samples from birds belonging to the zoological order Psittaciformes (generally referred to as parrots). The samples, collected over a period of seven years from November 2016 to September 2023, all originated from patients of a veterinary practice specialized and limited to parrots, living in Germany and the surrounding European region. A total of 14 different genera and 46 individual species were sampled.

Blood samples originated from leftover blood from different diagnostic workups of the individual birds in all cases. The birds were sampled due to various veterinary reasons, but not with the sole goal of obtaining HDL-C and LDL-C values. Reporting of this retrospective study followed the ARRIVE 2.0 guidelines [37]. Birds were fasted for at least 6 h prior to blood collection. To alleviate stress and potential suffering during the sampling process and other performed procedures, birds were placed under general anesthesia, induced with 5% isoflurane (Isofluran CP, CP-Pharma Handelsgesellschaft mbH, Burgdorf, Germany) via face mask in 2 L oxygen/minute. Right jugular venipuncture was performed by standard measure [37] using a 26-gauge, 12 mm needle attached to a 2 mL Syringe. The blood was collected into Serum Gel tubes (Sarstedt AG & Co. KG, Nümbrecht, Germany) and centrifuged 30 min after sampling. The serum was collected, cooled at 4 °C, and tested at Antech Lab Augsburg, Germany (previously Synlab Holding, Augsburg, Germany) on an Alinity ci-series c-module (Abbott, Abbott Park, IL, USA). TC was measured photometrically after separation of cholesteryl esters into free cholesterol and fatty acids. For the HDL-C test, non-HDL-C lipoproteins were combined with polyanions and a detergent to form a water-soluble complex, which was determined using the same enzymatic colorimetric method. Direct determination of LDL-C, where the same general principles were applied, was performed by the addition of a sugar compound and a detergent that selectively solubilizes LDL. Non-HDL-C was calculated by subtracting HDL-C from TC (Non-HDL-C = TC–HDL-C).

Additional information considered from oral anamnesis and physical examination of the birds included genus, species, age (if known), diet, body condition score, breeding condition, and current reproductive status. Some patients presented at the practice for routine check-ups and consequently had their blood sampled, HDL-C and LDL-C measured, and were included in this study more than once. Hence, the 1199 blood samples originate from a total of 692 subjects consisting of 249 parrots from the *Amazona* genus, 88 from the genera *Ara* and *Anodorhynchus*, 18 from the genus *Pionites*, 8 *Pionus*, 229 *Psittacus*, 19 *Poicephalus*, 23 *Eclectus*, 3 *Aratinga*, 2 *Deroptyus*, and 53 subjects from the family Cacatuidae [38]. The sample pool for the genera of *Aratinga*, *Deroptyus*, and *Pionus* were deemed too small for any quantitative analysis, therefore these birds were fully excluded from further evaluation.

### 2.2. Categorization of Variables

Sex was unrelated to this study, determined by DNA-sexing (PCR) from blood or feather, surgical sexing via endoscopy, post-mortem examination, or dimorphism in select species. Data were based on observations from 370 males and 322 females. The age of individuals ranged from 1 to 56 years. The birds’ diet was recorded and categorized in three categories. Category 1 included birds fed exclusively on seed mixtures, category 2 consisted of birds fed a mixture of seeds, food from table, and some pellets or extrudates. Category 3 consisted of birds exclusively fed a pelleted or extruded diet, with the majority of the birds in this category fed Harrison’s bird foods High Potency or Adult Lifetime formulas (Harrison’s bird foods, Brentwood, TN, USA). The intake of fruits and vegetables was not included in this variable, due to a wide range of variations. During physical examination, a BCS of the individual birds was recorded from 1–5, with 1 being cachectic, 3 being normal, and 5 being obese [39]. The variable of breeding condition was classified as a summary of the bird’s reproductive history, with three different categories. Category 1 included sexually immature, juvenile birds with no reproductive history. Category 2 included adult, sexually mature birds that have gone through breeding seasons and experienced the hormonal changes that come with them. Category 3 consisted of female parrots that have laid eggs in the past, regardless of them being fertilized or not. Breeding condition as a long-term variable was deemed suitable to be analyzed in relation to atherosclerosis, a slowly progressing disease, rather than lipoproteins. On the other hand, the variable of reproductive status defined the bird’s current reproductive activity at the time of sample collection fit for lipoprotein evaluation, which was grouped in four different categories. This evaluation was made based on seasonal behavioral changes, copulation, changes to the birds’ cloaca under the influence of estrogen, and endoscopic evaluation of the bird’s gonads [7]. Category 1 included juvenile or reproductively inactive birds, due to factors like age or disease. Category 2 included adult birds outside of breeding season, showing no signs of reproductive behavior, whereas birds that were under hormonal influence during the breeding season and exhibited said behavior were put into category 3. Lastly, category 4 was a category exclusively for adult, female parrots that laid eggs within one week before or after the time of sample collection. A summary table of the defined categories can be found in Supplementary File S1.

Whenever available, an additional screening for atherosclerosis was included as a variable, either by endoscopic evaluation (*n* = 872) of the large heart vessels and aorta descendens in living birds or post-mortem gross pathological examination in deceased birds (*n* = 14) [7,17,40]. Endoscopic evaluation was performed at the time of sample collection by the same person in all birds. In some cases, when repeated blood sampling occurred, diagnosis of atherosclerosis from previous examinations was transferred to later sample collections, as atherosclerotic lesions do not resolve. Due to a lack of an existing classification system for endoscopic evaluation, the observations were grouped into four different categories, either with no data available, no atherosclerosis observed, mild atherosclerosis, or moderate to severe atherosclerosis. The lesions observed during endoscopy or post-mortem examination were classified based on the size, overall number, artery rigidity, and color intensity of the whitish-yellow discoloration of the arterial plaques (Supplementary File S2). An endoscopic evaluation of the aorta descendens can be found in Supplementary Video S1.

All birds were evaluated as healthy or sick based on physical examination and individually available laboratory test results. Due to all birds being pets or kept in breeding facilities, no generalized categorization for ambient temperature, air humidity, or photoperiod was performed. The same is applicable for exercise and ability of flight, due to the huge differences and the lack of an existing categorization model for exercise.

### 2.3. Data Subsets

For establishing the reference intervals, only clinically healthy birds without signs of disease complexes were included, to ensure a healthy reference population. Additionally, birds with diagnosed non-clinically relevant atherosclerosis were excluded from the reference population. Exclusion criteria, in general, varied greatly due to the variance of health problems in parrots, but included, among others, anemia, leukocytosis, obesity, cachexia, pneumonia, cardiovascular disease, hepatopathy, nephropathy, reproductive disease, egg laying, and neoplasia. This left the population for reference intervals with 761 observations from 419 parrots in total, consisting of 215 males and 204 females, which was then fit to include only the first eligible observation from each bird. Thus, only healthy birds with one measurement each were included to determine the reference intervals. Due to the low amount of blood available for testing, only HDL-C or LDL-C were measured in a few cases, resulting in slightly differing reference populations between HDL-C, LDL-C, and non-HDL-C.

For the second aim of this study, for the evaluation of changes in HDL-C, LDL-C, and non-HDL-C values in dependence on different variables, all birds with existing HDL-C, LDL-C, and non-HDL-C values, regardless of health status, were included. However, 313 observations with a missing diagnosis concerning atherosclerosis were excluded. Further, 14 observations with missing values in either one of the independent or dependent variables included in the four developed models for HDL-C, LDL-C, non-HDL-C, and atherosclerosis, were excluded as well. This left 853 observations from 501 birds as the sample base for estimating the regression models.

For the third aim of this study, analyzing potential risk factors for atherosclerosis, a subset of the data without repeated measurements was used, to ease convergence of the model estimation. One subject was excluded from the analysis as it developed atherosclerosis during the repeated measurements. Furthermore, since none of the 12 *Pionites* birds exhibited atherosclerosis, all birds belonging to this genus were excluded from the analysis. This left the analysis with 488 birds and one observation each. A detailed overview showing the process of exclusion and how many birds were included in each model is stated in Supplementary Figure S1.

### 2.4. Data Analysis

All statistical analyses were conducted using R (v4.4.2; R Core Team 2024). For obtaining reference intervals (RI) for HDL-C, LDL-C, and non-HDL-C for each genus, ASVCP guidelines [41] were followed and the R package referenceIntervals (v1.3.1) [42] was used. Data from the previously described reference subset of the base population without repeated measures were used. Histograms for HDL-C, LDL-C, and non-HDL-C within all genera can be found in the Supplementary Information (Supplementary Figures S2–S4). Visual inspection of the histograms (Supplementary Figures S2–S4), together with results from Shapiro–Wilk tests (*p* < 0.05 for most genus–parameter combinations, with only four exceptions), indicated that the assumption of normality was generally not met. In a few cases, normality was not formally rejected; however, no genus showed normally distributed values across all blood parameters. To ensure methodological consistency and comparability across genera and blood values, we therefore used distribution-free methods for all reference interval estimations. For genera with more than 119 observations, namely *Amazona* and *Psittacus*, the empirical 2.5th and 97.5th quantiles were estimated using the nonparametric method implemented in the refLimit function of the previously mentioned R package. For taxa with 20 to 199 observations, namely Cacatuidae and *Ara*/*Anodorhynchus*, the recommended robust method [43] was used. For all RI limits, 90% confidence intervals (CI) were obtained via bootstrapping [44]. For genera with less than 20 observations, namely *Eclectus*, *Pionites*, and *Poicephalus*, individual observations and the median per genus per blood value can be found in the Supplementary Information (Supplementary Table S1). Further, after common tests for outlier detection, as proposed by the ASVCP guidelines, were deemed not suited due to sample sizes and distributional characteristics, the two highest and two lowest values were removed from each reference interval.

To further investigate influences on HDL-C, LDL-C, and non-HDL-C values, three linear mixed-effects models (LMM) were constructed, one for each blood value, and applied using the linear mixed-effects regression (lmer) function of the lme4 package (v1.1.35.5). All three models incorporated several independent variables, namely genus, age, sex, level of atherosclerosis, diet, current reproductive status, and the body condition score. Reference levels for the categorical variables can be found in the reported tables containing the resulting coefficient estimates (Supplementary Tables S2–S5). Additionally, random intercepts were included to account for individual variations among the birds. To assess the overall significance of the independent variables, Type II tests were calculated on the estimated models using the car package in R (v3.1.3) [45].

For investigating potential risk factors for atherosclerosis, the outcome variable was transformed into a binary variable indicating the presence or absence of atherosclerosis in birds. This transformation implies the application of a logistic Generalized Linear Model (GLM) to the data, here without using repeated measures. This was conducted using the glm function of the stats package (v4.4.2). The independent variables genus, age, sex, diet, breeding condition, and HDL-C, LDL-C, and non-HDL-C levels were included in the GLM. As for the three LMM for HDL-C, LDL-C, and non-HDL-C, a Type II test was conducted to estimate the overall significance of the independent variables. An alpha level of 0.05 was chosen as the threshold for statistical significance. The code and data used to conduct the analyses described can be found in Supplementary Files S3 and S4.

## 3. Results

### 3.1. Reference Ranges of HDL-C, LDL-C, and Non-HDL-C

Blood concentrations for HDL-C, LDL-C, and non-HDL-C obtained from the parrots included in this study are summarized in Table 1, Table 2 and Table 3. Genus-related differences were clearly visible, meaning that different genera of parrots have different reference intervals of HDL-C, LDL-C, and non-HDL-C and need to be evaluated accordingly in practice. The lowest median HDL-C values were recorded in macaws (genera *Ara* and *Anodorhynchus*), the highest in *Eclectus* parrots, followed by grey parrots (*Psittacus*) and Amazon parrots (*Amazona*). For LDL-C, the lowest median was again observed in macaws, whereas the highest was found in *Psittacus* and *Eclectus*. The lowest median non-HDL-C values were recorded in *Pionites*, closely followed by macaws, the highest in *Psittacus* followed by *Eclectus*.

### 3.2. HDL-C Model

**Figure 1:**
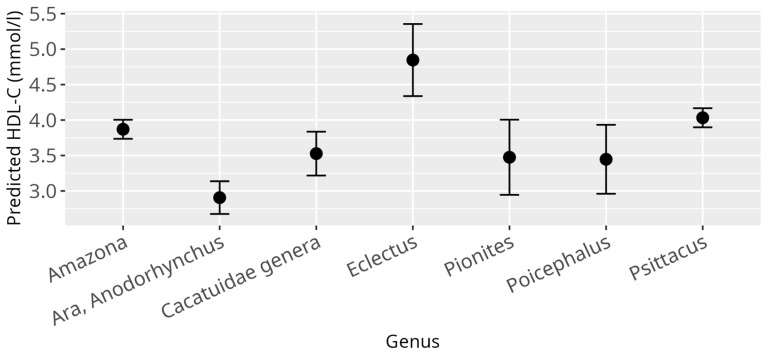
Effect plots display the predicted outcome values for each category or value of a covariate, with other variables held constant. Points indicate model predictions, and error bars represent 95% confidence intervals, seen here for HDL-C and genus; x-axis shows investigated psittacine genera; y-axis shows predicted HDL-C in mmol/L; the dot in the middle of the error bars displays the median.

**Figure 2:**
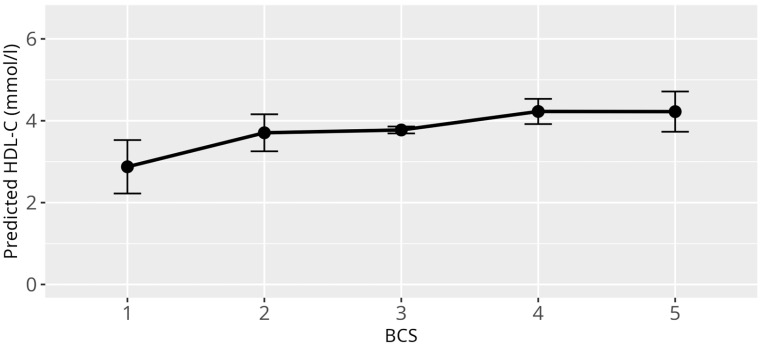
Effect plot with confidence intervals for HDL-C and BCS; x-axis shows BCS categories (1 = cachectic, 2 = reduced bodyweight, 3 = normal, 4 = increased bodyweight, 5 = obese); y-axis shows predicted HDL-C values in mmol/L; the dot in the middle of the error bars displays the median.

Within the following, all effect estimates reported were to be interpreted on average and all other variables in the according model were kept constant. Overall, the LMM applied reveals a significant relationship between HDL-C levels and several independent variables. Genus was found to be significantly associated with HDL-C levels (*p* < 0.001) (Figure 1), confirming the large differences between the genera of HDL-C values observed in the analyses of the empirical quantiles. The BCS also showed a significant effect on HDL-C levels (*p* = 0.002), with higher levels of BCS, BCS 4 (β = 0.45, SE = 0.16, *p* = 0.01), and 5 (β = 0.45, SE = 0.25, *p* = 0.08) being associated with increased HDL-C levels, and vice versa for the lowest BCS (β = −0.90, SE = 0.33, *p* = 0.01) being associated with lower HDL-C levels, as compared to HDL-C levels corresponding to a healthy BCS (Figure 2). No significant associations between HDL-C levels and age, sex, diet, reproductive status, and atherosclerosis prevalence were observed (Supplementary Figure S2 and Supplementary Table S1).

### 3.3. LDL-C Model

**Figure 3:**
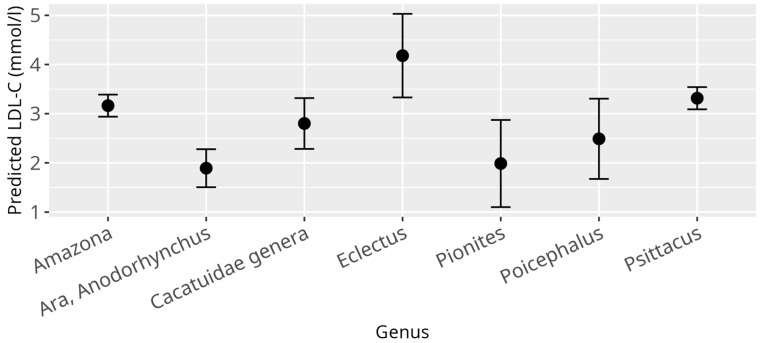
Effect plot with confidence intervals for LDL-C and genus; x-axis shows investigated psittacine genera; y-axis shows predicted LDL-C in mmol/L; the dot in the middle of the error bars displays the median.

**Figure 4:**
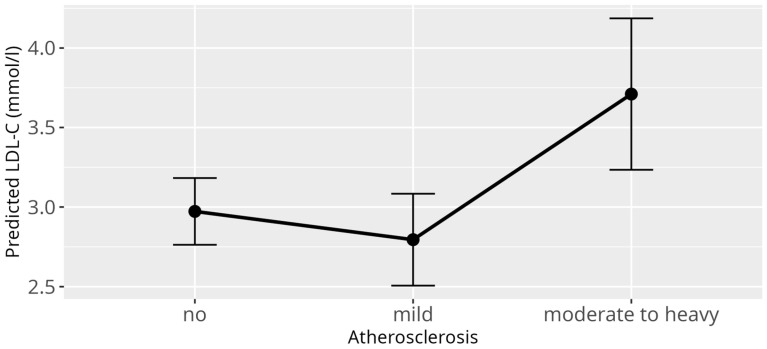
Effect plot with confidence intervals for LDL-C and atherosclerosis; x-axis shows atherosclerosis severity; y-axis shows predicted LDL-C values in mmol/L; the dot in the middle of the error bars displays the median.

**Figure 5:**
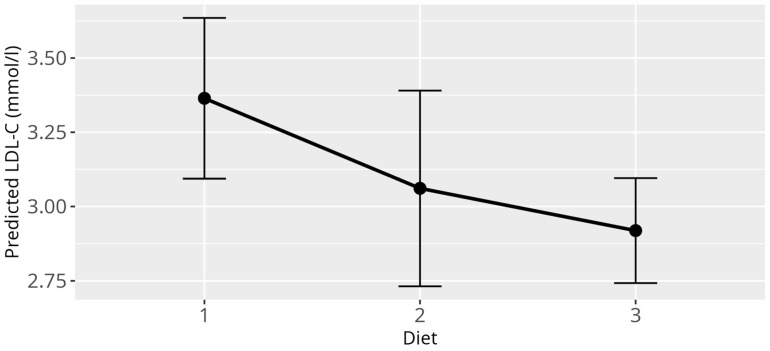
Effect plot with confidence intervals for LDL-C and diet; x-axis shows dietary categories (category 1 pure seed diet; category 2 mixed diet seeds, table food, and pellets/extrudates; category 3 pure pellets/extrudates); y-axis shows predicted LDL-C in mmol/L; the dot in the middle of the error bars displays the median.

When investigating influences on the LDL-C levels, several significant relations could be identified through application of the LMM. As it was in the case for HDL-C, there was a strong effect of the genera (*p* < 0.001) (Figure 3), confirming once again the large differences between birds from different genera as observed for the reference values. There was a significant relation between BCS (*p* = 0.03) and LDL-C values. The only significant effect on LDL-C within BCS, when compared to a healthy score, was observed for cachectic birds (β = 1.42, SE = 0.55, *p* = 0.01). However, it has to be stated, that for this data subset, only nine observations were available for cachectic birds from which one value represented an outlier (LDL-C = 15.95 mmol/L). Neither the overall effect of the BCS nor the effect of the cachectic sample on LDL-C remained when this outlier was removed (Supplementary Figures S3 and S4). Additionally, for LDL-C levels, atherosclerosis (*p* = 0.001) as well as the diet (*p* = 0.03) displayed significant overall effects (Figure 4 and Figure 5). More precisely, birds that were diagnosed with moderate to severe atherosclerosis (β = 0.74, SE = 0.30, *p* = 0.01) seemed to have increased levels of LDL-C as compared to birds without atherosclerosis. This effect was not observed in birds diagnosed with mild atherosclerosis (β = −0.18, SE = 0.20, *p* = 0.37). Further, birds exclusively fed a pelleted or extruded diet (category 3) (β = −0.45, SE = 0.17, *p* = 0.01) displayed significantly lower LDL-C values as compared to birds exclusively fed on seeds (category 1), while there is no significant difference between birds from category 1 and birds fed a mixture of seeds, food from table, and pellets or extrudates (category 2) (β = −0.30, SE = 0.21, *p* = 0.16). There appeared to be a significant relation between reproductive status and LDL-C (*p* = 0.01), with the group of adult female parrots that laid eggs within one week before or after the time of sample collection (category 4), consisting of 10 animals, driving this main effect (β = −1.69, SE = 0.61, *p* = 0.01).

### 3.4. Non-HDL-C Model

**Figure 6:**
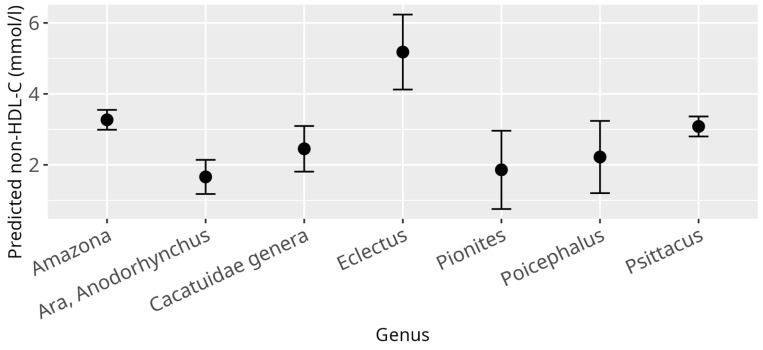
Effect plot with confidence intervals for non-HDL-C and genus; x-axis shows investigated psittacine genera; y-axis shows predicted non-HDL-C in mmol/L; the dot in the middle of the error bars displays the median.

**Figure 7:**
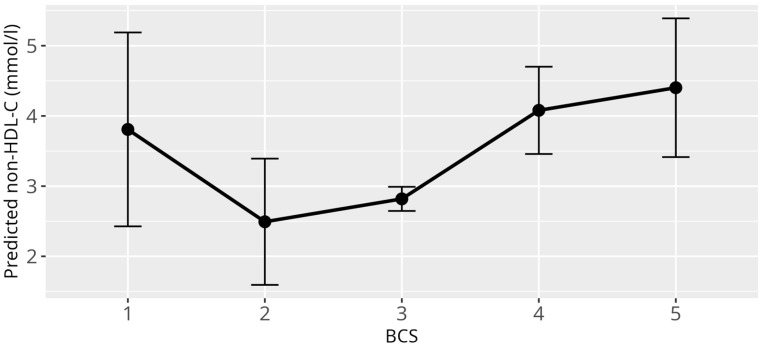
Effect plot with confidence intervals for non-HDL-C and BCS; x-axis shows BCS categories (1 = cachectic, 2 = reduced bodyweight, 3 = normal, 4 = increased bodyweight, 5 = obese); y-axis shows predicted non-HDL-C values in mmol/L; the dot in the middle of the error bars displays the median.

**Figure 8:**
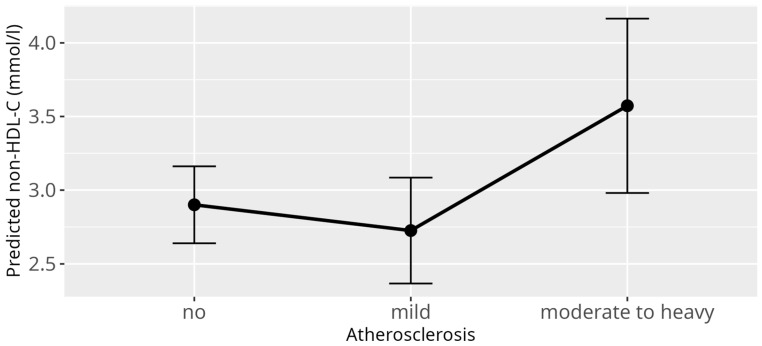
Effect plot with confidence intervals for non-HDL-C and atherosclerosis; x-axis shows atherosclerosis severity; y-axis shows predicted non-HDL-C values in mmol/L; the dot in the middle of the error bars displays the median.

Applying the previously described linear mixed model (LMM) with non-HDL-C levels as the response variable identified several significant associations. Consistent with the HDL-C and LDL-C models, a strong difference was observed between genera (*p* < 0.001) (Figure 6). Additionally, atherosclerosis exhibited a significant overall effect (*p* = 0.03), while both BCS (*p* < 0.001) and reproductive status (*p* < 0.001) showed highly significant overall effects. Specifically, cachectic birds with BCS 1/5 (β = 2.22, SE = 0.68, *p* = 0.001), and birds with increased bodyweight classified as BCS 4/5 birds (β = 1.23, SE = 0.33, *p* < 0.001), and obese birds with BCS 5/5 (β = 1.54, SE = 0.52, *p* = 0.001) had significantly higher non-HDL-C values compared to birds with a normal BCS 3/5 (Figure 7). Similar to the LDL-C model, an outlier was detected among the nine cachectic birds, with one individual displaying exceptionally high cholesterol levels (20.49 mmol/L), justifying its classification as an outlier. When this observation was excluded and the model was refitted, the overall effect of atherosclerosis was reduced to a trend (*p* = 0.06) (Figure 8), while the effect of reproductive status remained highly significant (*p* < 0.001). As with the LDL-C model, the main effect of reproductive status appears to be primarily driven by adult female parrots that laid eggs within one week before or after sample collection (β = 3.61, SE = 0.75, *p* < 0.001), while an additional effect could be shown for adult birds during breeding season, that showed signs of reproductive behavior (β = 0.48, SE = 0.22, *p* = 0.05). No significant associations between non-HDL-C levels and age, sex, and diet were observed.

### 3.5. Atherosclerosis Model

**Figure 9:**
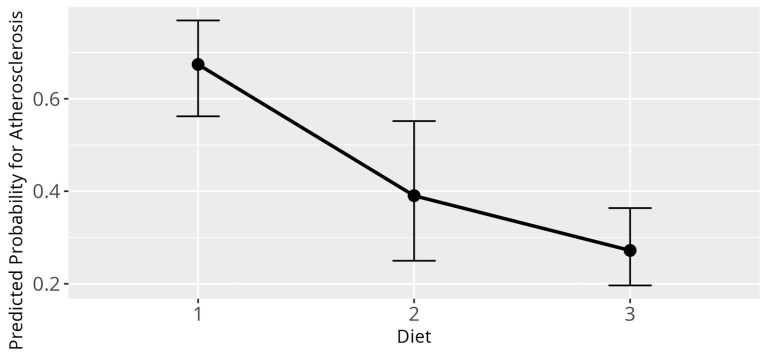
Effect plot with confidence intervals for atherosclerosis and diet; x-axis shows dietary categories (category 1 pure seed diet; category 2 mixed diet seeds, table food, and pellets/extrudates; category 3 pure pellets/extrudates); y-axis shows predicted probability for atherosclerosis; the dot in the middle of the error bars displays the median.

**Figure 10:**
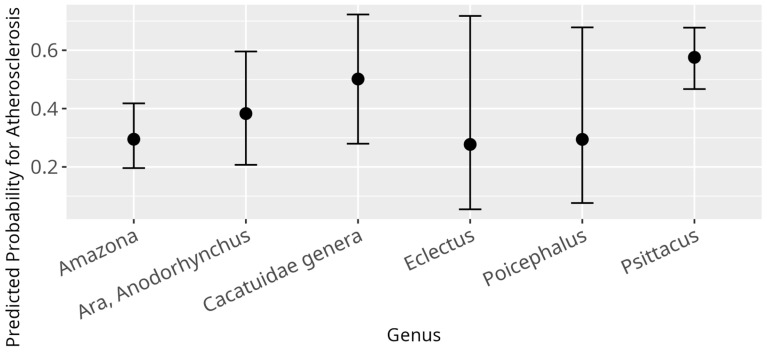
Effect plot with confidence intervals for atherosclerosis and genus; x-axis showing investigated genera; y-axis showing the predicted probability for atherosclerosis; the dot in the middle of the error bars displays the median.

The GLM, modelling influences of various variables on the odds to be diagnosed with atherosclerosis, displayed several significant relations. Firstly, for a one-year rise in age, the odds of being diagnosed with atherosclerosis rose by a factor of 1.18 (β = 0.16, SE = 0.02, *p* < 0.001). Further, diet had a significant overall influence (*p* < 0.001), with birds fed according to category 2 (β = −1.17, SE = 0.41, *p* = 0.004) as well as birds fed according to category 3 (β = −1.71, SE = 0.32, *p* < 0.001) displaying lower probability for atherosclerosis as compared to birds fed according to category 1 (Figure 9). Additionally, a significant difference between the genera was found (*p* = 0.01) (Figure 10). No significant correlation between atherosclerosis and the variables sex, HDL-C, LDL-C, non-HDL-C, BCS, and breeding condition were observed.

## 4. Discussion

The results of this study present large-scale, multi-genera reference intervals for HDL-C, LDL-C, and non-HDL-C in pet parrots. The analysis of reference intervals revealed significant differences associated with the genus. HDL-C, LDL-C, and non-HDL-C values differed between genera and need to be interpreted accordingly. The genus *Ara*, including *Anodorhynchus*, which in the past have been reported to be the least susceptible genus to atherosclerosis [9], showed the lowest levels of HDL-C and LDL-C, an observation that confirms results from a previous study [35]. The genus of *Psittacus*, which has been previously shown to be highly susceptible to atherosclerosis [9], showed consistently high median values for HDL-C, LDL-C, and non-HDL-C. The genus of *Eclectus*, also commonly observed with atherosclerosis, showed similarly high medians of HDL-C, LDL-C, and non-HDL-C; however, the incidence of atherosclerosis in Eclectus parrots has previously been suggested to be lower than in African grey parrots or Amazons [12,46]. Additionally, *Eclectus* as a genus is difficult to evaluate, due to the autapomorphy it possesses within the order of Psittaciformes. The genus demonstrates a unique physiology and biology, which is also reflected in its serum biochemistry values [2,38,47]. Consequently, high medians in HDL-C, LDL-C, and non-HDL-C in this genus may not adequately compare to other psittacines. In the author’s avian practice, *Eclectus* showed a prevalence and susceptibility for atherosclerosis similar to the genera *Psittacus* and *Amazona*. For the present study, the total number of HDL-C and LDL-C measures in *Eclectus* parrots were only *n* = 43 from *n* = 18 individual birds, compared to a much higher number in Amazons and African greys, so the results have to be interpreted with caution. More *Eclectus*-focused studies will be needed to determine the scale of their susceptibility to atherosclerosis and the correlation between high HDL-C, LDL-C, and non-HDL-C values.

In the GLM with atherosclerosis as a dependent variable, the only statistically significant genus-related difference was recorded for African grey parrots in relation to Amazon parrots. The genus *Psittacus* showed a higher predicted probability of developing atherosclerosis than the genus *Amazona*. For the other genera, no differences could be detected, and while this may be due to the fact that the aforementioned genera had the largest sample sizes in this study, the result of the high atherosclerosis susceptibility of the genus *Psittacus* was in line with observations from previous studies [9,48].

When studying the LMM for HDL-C, LDL-C, and non-HDL-C for their relation to atherosclerosis, a significant change in the predicted levels of LDL-C in the presence of moderate to severe atherosclerosis was observed. A similar trend was observed in the predicted levels of non-HDL-C in the presence of moderate to severe atherosclerosis, with a *p*-value lower than in the LDL-C model and just below the threshold for statistical significance at 0,06. However, the extent of the influence of the other encompassed lipoproteins in the non-HDL-C parameter, next to LDL-C, in parrots, remains unclear. Non-HDL-C also includes IDL, VLDL, and portomicrons, which have been suggested to be atherogenic in character as well, but might alter the results accordingly. In humans, VLDL has been demonstrated to be significantly less proatherogenic than LDL [49,50]. Contrary to observations in the LMM, in the GLM with atherosclerosis as a dependent variable, high levels of LDL-C, non-HDL-C, or HDL-C were not significant predictors of atherosclerosis. This, however, may be due to the change of the character of the variable from categorial in the LLM into binary in the GLM. Diagnoses of light atherosclerosis were mixed together with moderate to severe atherosclerosis, and potentially observable effects for stronger atherosclerosis may have been obscured. Consequently, it can be assumed that, while there is a correlation between the presence of moderate to severe atherosclerosis and a high LDL-C value, and in part a high non-HDL-C value, a high LDL-C or non-HDL-C value itself cannot reliably predict or diagnose atherosclerosis. When looking at this result, it makes sense that a high LDL-C or non-HDL-C value has to be interpreted as a potential risk factor and indicator for atherosclerosis, but not a definitive statement for the presence of atherosclerotic disease. Other than in a study conducted by Beaufrère et al. [35], where a potential connection between atherosclerosis and a high HDL-C was presumed, our results indicate a correlation between atherosclerosis prevalence and increasing LDL-C and non-HDL-C, rather than HDL-C. HDL-C showed no statistically significant relation to atherosclerosis prevalence in the present study, contrary to our hypothesis. This result aligns with studies conducted in human medicine, where LDL-C has been identified as a major prognostic factor in the development and further evolution of atherosclerosis and cardiovascular disease. Naturally, the extrapolation of scientific findings from humans to birds has to be interpreted with caution due to the considerable species barrier, even though animal models play a crucial part in studying the pathomechanisms of atherosclerosis [21]. In human medicine, mendelian randomization studies and randomized controlled trials consistently demonstrated a log-linear relationship between the risk of atherosclerotic cardiovascular diseases (ASCVD) and the absolute changes in plasma LDL-C [22,23,51,52,53]. Biological and experimental evidence, in addition to the consistency among these studies, provides compelling evidence that LDL-C is causally associated with the risk of ASCVD in humans. Contrary to that, mendelian randomization studies about HDL-C do not provide compelling evidence that HDL-C is causally associated with the risk of ASCVD [52,54,55]. There is much rather an inverse association between HDL-C and the risk of ASCVD [23], which continues to be subject of discussion. In chickens, HDL deficiency syndrome did not result in increased susceptibility to atherosclerosis [56]. In another study, chickens on an atherogenic high-cholesterol, high-triglyceride diet had increased carotenoid, cholesterol, and protein content in the LDL fractions, but not the HDL fractions [57]. In reptiles, atherosclerosis is scarcely reported and consequently poorly understood [58]. However, when present, LDL-C has been speculated to be the most prominent risk factor for its development [58,59].

A study on lipoprotein cholesterol concentrations in 31 parrots of the genus *Amazona* [36] found no significant correlation between lipoprotein cholesterol concentrations and age (younger or older than 20 years), sex, and diet, apart from a difference in HDL-C concentration in male and female Amazons, the latter which we were not able to confirm in our study. Our results further suggested that BCS in parrots seems to be connected to HDL-C and non-HDL-C levels, but not to LDL-C levels.

While we confirmed that there was no connection between HDL-C and diet, there appeared to be a significant correlation between LDL-C and diet, like it was hypothesized. Seed diets in comparison to pelleted or extruded diets have been shown to contain excess fat, low calcium to phosphorus ratios, and many other deficiencies in vitamins and minerals [60,61,62]. Birds fed a balanced diet of extrudates or pellets showed significantly lower LDL-C values than birds on a mixture of different foods or a pure seed diet. Interestingly, this was not observable for non-HDL-C, which showed no connection to the respective dietary categories. This again raises the question of how the other lipoproteins encompassed in non-HDL-C, next to LDL-C, alter the effect, or if this is perhaps attributed to the potentially inaccurate, direct measurement of LDL-C.

At the same time, diet had an overall influence on the prevalence of atherosclerosis, observed in the GLM. Birds on a diet from categories 2 and 3 reliably displayed lower probability for the development of atherosclerosis as compared to the birds that were on a pure, unbalanced seed diet. Diet-induced atherosclerosis has been demonstrated experimentally in budgerigars (*Melopsittacus undulatus*) and Quaker parrots (*Myiopsitta monachus*) [34,63]. Quaker parrots fed a 1% cholesterol diet developed severe dyslipidemias with marked increases of TC and LDL-C and advanced atherosclerotic lesions in the aorta, brachiocephalic trunks, and coronary arteries within 4 months [34]. TC by itself, however, has proven to be an inadequate marker for the diagnosis and development of atherosclerosis, and in clinical situations, the single evaluation of TC in regards of diagnostics for atherosclerosis lacks significance. Birds have been observed with atherosclerosis, but normal TC values; however, parrots with high TC and the absence of atherogenic changes have been commonly reported as well [64,65]. In addition, female parrots undergoing seasonal reproductive activity and vitellogenesis showed large-scale elevations of TC and triglycerides among other biochemical parameters without vascular disease being present, further complicating the evaluation of TC [66,67].

In this study, contrary to our hypothesis, sex had no significant effect on neither HDL-C, LDL-C, and non-HDL-C concentrations, nor on the prevalence of atherosclerosis. Other studies on lipoproteins in psittacines were inconsistent regarding sex differences for lipids too. While some also showed no difference [35], others marked elevated HDL-C levels in females compared to males [36] or higher LDL-C in females [68]. Surprisingly, in this study, reproductive status at time of sample collection showed no effect on HDL-C, LDL-C, and non-HDL-C values, apart from a significance for LDL-C and non-HDL-C for females that reportedly laid an egg within one week before or after sample collection (category 4). Additionally, an effect for non-HDL-C was demonstrated for adult birds during breeding season, that showed signs of reproductive behavior (category 3), hinting at changes to the lipoprotein profile during breeding season. Due to the small number of observations in category 4, this significance is likely to be attributed to an instability of the models. In the event of a confirmation of this effect with a larger sample size, these changes would likely relate to the effects of estrogens on the lipid metabolism, as well as protein and calcium metabolism in reproductively active females. Estrogen promotes increased plasma TC, TG, calcium, and protein levels [69]. These changes may promote atherogenesis and may at least partially explain the association between reproductive tract disease and increased prevalence of advanced atherosclerotic lesions [9]. Breeding condition was studied for its correlation with the presence of atherosclerosis as well, but showed no significant effect in that matter. While there was an expected correlation with age, from an evolutionary perspective, it makes sense that seasonal reproductive behavior would show no immediate effect on the presence of atherosclerosis, as this would be counterproductive to the continued existence of a species, if the majority of their individuals developed marked disease after reproducing multiple times.

The present study results showed that age had no significant effect on HDL-C, LDL-C, and non-HDL-C levels, which was in line with another study on its influence on the development of atherosclerosis [35]. However, there was a strong association between rising age and the development of atherosclerosis observed in the GLM, reconfirming results from previous studies [9]. As atherosclerosis is characteristically a slowly progressing disease, it makes sense that with increasing age, there will be more individuals with the disease present. It is likely that, as in humans, all birds will eventually develop some degree of atherosclerotic disease with rising age, which may or may not be clinically distinct and actually cause symptoms. At that point, the individual needs to be evaluated for other present risk factors and overall health status, so actual therapy and preventive measures can be applied.

While LDL-C and non-HDL-C significantly differed in their actual values in this study, they showed similar changes in dependence of atherosclerosis severity. Observed effects for LDL-C and diet could not be demonstrated for non-HDL-C. It remains to be determined if these differences are due to the fact that LDL-C may be overestimated in the direct measurement on a biochemistry analyzer or if other lipoproteins next to LDL-C encompassed in non-HDL-C obscured the effect. Future studies should try to document and compare the effects in additional diagnostic modalities like lipoprotein electrophoresis and HPLC.

There are some limitations within this study and the reference intervals established. A significant limitation is found due to the fact that all parrots in this study were pets or breeding birds and originated from different places, where they were kept under different settings. While this variability renders the sample pool inhomogeneous, it concurrently offers a representative cross-section of bird species commonly encountered in daily avian practice. Though avoiding exercise and a sedentary lifestyle is another known risk factor for atherosclerosis in humans, the lack of a classifiable scale and unequal husbandry conditions made an evaluation impossible and for this reason was excluded from this study. The same is applicable for genetics. Genetic influences on serum lipoprotein levels in humans have been studied for many years now, and a reference study from as early as 1990 demonstrated that the proposed genetic locus accountable for LDL-C subfraction phenotypes eventually results in an atherogenic lipoprotein phenotype [70]. Genetic disorders, like familial hypercholesterolemia, probably the most common monogenic dyslipidemia, can cause premature atherosclerosis and cardiovascular complications [71]. However, while hypercholesterolemia is common in psittacine birds [67,72], avian medicine lacks identified genetic disorders for parrots so far, and though some of the parrots in this study might be affected, they consequently fell under the radar. The classification system used for categorizing the variables of breeding status and reproductive status are experimental. These variables are difficult to classify and the system used may not represent the actual shifts to the lipoprotein profile during the different reproductive states.

Another limitation to this study is the method for determining the HDL-C and LDL-C values, which in this study were measured on a classic biochemistry analyzer as direct photometrical measurement using an enzymatic, colorimetric test, rather than on a more accurate, but less available and high cost, HPLC. HDL-C has been proven to be very accurate, when measured on a biochemistry analyzer, and while LDL-C has been shown more difficult to properly measure [31], it, at the moment, is the most convenient way to obtain direct LDL-C values. The Friedewald formula is another commonly used equation and indirect method to calculate LDL-C values in humans, but has shown to be inaccurate for parrots, especially in lipemic samples [31,67]. General effects of lipemia on measurements of cholesterols in parrots are, however, largely unknown so far. In this study, only HDL-C and LDL-C without their existing subclasses were evaluated. While today it is clear that specific subclasses of LDL-C, like the small-dense-LDL-C, are more atherogenic than others [73], pilot studies and base intervals for the main groups of HDL-C and LDL-C had to be performed first, before subfractions can be evaluated.

Because of lacking standard procedures and the available methodologic options, occurrence of atherosclerosis within the present study was primarily diagnosed by endoscopy in living birds and gross pathology in deceased birds. X-ray, a commonly used tool in avian practice, which has additionally been applied in some birds of this study, has been shown to have a low sensitivity when it comes to diagnosing atherosclerosis [6,14], only being able to reliably diagnose birds with severe detectable calcification of the great vessels. Endoscopy, though an invasive procedure, is a useful tool to identify various diseases in pet birds [74] and has been described to be used for the diagnosis of atherosclerosis in parrots before [7,17,40,75]. For this study, the large heart vessels and aorta descendens were evaluated endoscopically. However, assessments have, up until now, never been cross checked with histologically based investigations. The results of this subjective evaluation may differ from the actual extent of the lesions. Additionally, lesions on vessels not visible in our investigation remained undetected, which is why identification of atherosclerotic birds in this study has to be interpreted with some caution. Further studies on the value of this invasive endoscopic method for diagnosing atherosclerosis will be needed in the future. The outcomes of post-mortem examinations were also used for diagnosis of atherosclerotic disease. Post-mortems provide the best results for identifying atherosclerotic lesions, and offer insight into the extent of the disease in retrospect [6,9]. Additionally, it allowed the subjective comparison of actual results with ante-mortem endoscopic diagnostics, which in this study came to the same conclusions. However, since all birds in this study were pets or birds in breeding facilities, the total number of cases diagnosed in this more accurate post-mortem method was comparatively low.

## 5. Conclusions

In conclusion, the lipoprotein panel has the potential to become a screening tool for clinicians in the diagnosis of dyslipidemias. This study provides new insight on the value of HDL-C and LDL-C in parrots and provides reference intervals for direct measurement of HDL-C, LDL-C on a biochemistry analyzer, and consequent calculation of non-HDL-C in many different genera of psittacines. Next to prominent genus-related differences for HDL-C, non-HDL-C, and LDL-C, the latter has shown to be elevated in the presence of moderate to severe atherosclerotic disease and is influenced by diet. A similar, but slightly less significant, trend was observed for non-HDL-C, which was elevated in the presence of moderate to severe atherosclerotic disease. However, these parameters are not a sole indicator of the presence of atherosclerotic disease and should rather be interpreted as a risk factor alongside variables like age and diet, which have also been shown to be associated with atherosclerosis. The clinician should therefore put special focus on lipoproteins (LDL-C and non-HDL-C), diet (seed diets), and genus (*Psittacus*), when it comes to the individual risk of a parrot developing atherosclerotic disease. However, further studies will be needed, to more precisely define the link between lipoproteins and atherosclerosis in parrots.

## Figures and Tables

**Table 1:** Reference intervals for HDL-C blood concentrations (mmol/L) in selected psittacine genera.

Genus	Sample Size	RI Method ^1^	RI ^2^	Median	CI ^3^ Lower RI	CI ^3^ Upper RI	Min ^4^	Max ^4^
*Amazona*	144	*n*	[2.53, 6.36]	3.70	[2.32, 2.65]	[6.01, 6.78]	2.41	6.99
*Psittacus*	122	*n*	[2.93, 5.28]	3.89	[2.81, 2.97]	[4.93, 5.50]	2.90	5.65
*Ara* and *Anodorhynchus*	64	r	[1.53, 3.97]	2.81	[1.30, 1.72]	[3.78, 4.18]	1.53	3.76
Cacatuidae genera	24	r	[1.39, 5.67]	3.36	[0.67, 1.96]	[5.05, 6.50]	1.89	5.96

^1^ RI (reference interval) method indicates the statistical method used for RI determination: *n* = nonparametric method (for sample sizes ≥ 120), r = robust method (for sample sizes < 120). ^2^ RIs are presented as the central 95% interval (2.5th to 97.5th percentiles) with corresponding 90% confidence intervals (CIs) for the lower and upper RI limits shown in brackets. ^3^ CIs were calculated using the bootstrapping method. ^4^ Min and Max represent the smallest and largest values observed in the sample data for each genus.

**Table 2:** Reference intervals for LDL-C blood concentrations (mmol/L) in selected psittacine genera.

Genus	Sample Size	RI Method ^1^	RI ^1^	Median	CI ^1^ Lower RI	CI ^1^ Upper RI	Min ^1^	Max ^1^
*Amazona*	144	*n*	[1.19, 7.99]	2.86	[1.06, 1.28]	[6.86, 9.52]	1.09	10.54
*Psittacus*	122	*n*	[1.54, 5.70]	3.37	[1.22, 1.66]	[5.43, 6.24]	1.42	5.96
*Ara* and *Anodorhynchus*	58	r	[0.46, 3.29]	2.00	[0.19, 0.67]	[3.08, 3.55]	0.67	3.21
Cacatuidae genera	24	r	[1.03, 5.26]	3.12	[0.44, 1.52]	[4.68, 5.88]	1.42	5.34

^1^ A description of values displayed can be found in the footer of Table 1.

**Table 3:** Reference intervals for non-HDL-C blood concentrations (mmol/L) in selected psittacine genera.

Genus	Sample Size	RI Method ^1^	RI ^1^	Median	CI ^1^ Lower RI	CI ^1^ Upper RI	Min ^1^	Max ^1^
*Amazona*	143	*n*	[0.93, 9.52]	2.48	[0.77, 1.17]	[6.40, 12.21]	0.52	15.61
*Psittacus*	122	*n*	[1.51, 6.27]	2.87	[1.21, 1.57]	[3.64, 8.01]	1.45	9.09
*Ara* and *Anodorhynchus*	62	r	[−0.02, 2.86]	1.52	[−0.35, 0.28]	[2.55, 3.19]	0.34	4.07
Cacatuidae genera	24	r	[0.26, 3.96]	2.11	[−0.21, 0.75]	[3.32, 4.57]	1.09	3.99

^1^ A description of values displayed can be found in the footer of Table 1.

## Data Availability

The original data presented in the study are openly available in the Supplementary Materials.

## Supplementary Materials

The following supporting information can be downloaded at https://www.mdpi.com/article/10.3390/ani15172493/s1; File S1: Definition and categorization of investigated independent variables. File S2: Grading system used for endoscopic diagnosis and evaluation of atherosclerosis in psittacines. File S3: R-Code and instructions for running. File S4: Dataset. Table S1: Reference values for genera Poicephalus, Pionites, and Eclectus. Due to sample size, these genera are not eligible for reference interval (RI) calculation. Each row within each genus represents one animal. Table S2: β, confidence intervals (CI) and *p*-values for coefficient estimates of covariates in HDL-C model. Reference categories are no atherosclerosis, Amazona, BCS of 3, male, fed according to category 1, and the reproductive status of category 1. Table S3: β, confidence intervals (CI), and *p*-values for coefficient estimates of covariates in LDL-C model. Reference categories are no atherosclerosis, Amazona, BCS of 3, male, fed according to category 1, and the reproductive status of category 1. Table S4: β, confidence intervals (CI), and *p*-values for coefficient estimates of covariates in non-HDL-C model. Reference categories are no atherosclerosis, Amazona, BCS of 3, male, fed according to category 1, and the reproductive status of category 1. Table S5: β, confidence intervals (CI), and *p*-values for coefficient estimates of covariates in atherosclerosis model. Reference categories are Amazona, BCS of 3, male, fed according to category 1, and the breeding status of category 1. Figure S1: Flowchart of regression populations and their exclusion process of birds. Figure S2: Histogram of HDL-C values within genera, additionally displaying the median within genera. Figure S3: Histogram of LDL-C values within genera, additionally displaying the median within genera. Figure S4: Histogram of non-HDL-C values within genera, additionally displaying the median within genera. Figure S5: Effect plot with confidence intervals for HDL-C and atherosclerosis. Figure S6: Effect plot with confidence intervals for LDL-C and BCS with outlier. Figure S7: Effect plot with confidence intervals for LDL-C and BCS without outlier. Figure S8: Effect plot with confidence intervals for non-HDL-C and BCS with outlier. Figure S9: Effect plot with confidence intervals for non-HDL-C and BCS without outlier. Figure S10: Mosaic plot of age and atherosclerosis. X-axis giving age in years in categories 1–10, 11–20, 21–25, 26–30, and >30; Y-axis indicating presence of atherosclerosis in yes and no. Figure S11: Mosaic plot of diet and atherosclerosis. X-axis giving diet in category 1 pure seed diet, category 2 mixed diet: seeds, table food, pellets/extrudates, category 3 pure pellets/extrudates; Y-axis indicating presence of atherosclerosis in yes and no. Video S1: Endoscopic diagnosis of atherosclerosis on aorta descendens and its branches in a 33-year-old African Grey parrot (Psittacus erithacus erithacus).
