# Supplementary material for: Serum Lipid Reference Intervals of High-Density, Low-Density and Non-High-Density Lipoprotein Cholesterols and Their Association with Atherosclerosis and Other Factors in Psittaciformes
Source: Animals (Basel). 2025 Aug 25;15(17):2493. doi: 10.3390/ani15172493 (PMC12427453; doi:10.3390/ani15172493)
Supplement: Supplementary file 1 [file animals-15-02493-s001.zip › animals-3796595-supplementary/Table S1.pdf]

**Poicephalus** (n = 15)			**Pionites** (n = 18)			**Eclectus** (n = 18)		
**LDL-C**	**HDL-C**	**Non-HDL-C**	**LDL-C**	**HDL-C**	**Non-HDL-C**	**LDL-C**	**HDL-C**	**Non-HDL-C**
2.15	1.17	3.88	1.99	3.70	2.05	0.67	2.36	11.60
1.81	2.12	1.58	2.49	2.77	2.41	4.22	4.20	1.76
2.05	2.02	1.79	2.98	3.65	2.85	3.03	3.37	3.03
3.73	4.38	2.64	1.81	2.95	1.35	3.13	4.53	2.39
2.38	3.99	2.36	1.61	1.79	0.80	3.00	4.14	2.52
1.42	3.16	1.71	2.38	2.75	1.39	2.85	4.01	2.70
2.82	3.57	2.75	1.27	3.06	1.27	2.72	4.25	2.43
3.19	3.83	3.01	0.83	3.91	1.01	2.07	5.26	1.40
3.26	3.21	3.01	0.88	3.83	0.62	3.08	8.16	7.04
3.08	3.78	1.92	1.22	3.52	1.40	3.44	3.89	2.71
4.40	4.56	2.95	2.10	2.80	1.55	3.32	4.35	2.82
2.15	2.59	1.68	2.31	3.52	0.88	1.94	5.13	3.21
1.61	3.99	2.46	NA	2.41	0.70	4.56	4.53	2.46
1.58	2.43	1.90	2.33	3.11	1.29	7.59	4.17	8.52
3.06	4.17	2.10	2.41	2.90	1.45	0.52	1.79	0.72
**2.38**	**3.57**	**2.36**	2.02	2.56	2.02	5.13	4.48	4.59
			3.26	3.81	3.11	19.04	4.90	24.52
			2.59	3.44	2.39	6.01	5.23	5.47
			**2.1**	**3.08**	**1.40**	**3.10**	**4.3**	**2.76**
