# Supplementary material for: Serum Lipid Reference Intervals of High-Density, Low-Density and Non-High-Density Lipoprotein Cholesterols and Their Association with Atherosclerosis and Other Factors in Psittaciformes
Source: Animals (Basel). 2025 Aug 25;15(17):2493. doi: 10.3390/ani15172493 (PMC12427453; doi:10.3390/ani15172493)
Supplement: Supplementary file 1 [file animals-15-02493-s001.zip › animals-3796595-supplementary/Table S2.pdf]

**Predictors**	β	**CI (2.5%)**	**CI (97.5%)**	**p**
(Intercept)	3.99	3.72	4.27	< 0.001
Atherosclerosis (mild)	0.08	-0.15	0.31	0.51
Atherosclerosis (moderate to heavy)	0.15	-0.20	0.50	0.41
Genus (Ara and Anodorhynchus)	-0.96	-1.23	-0.70	< 0.001
Genus (Cacatua)	-0.34	-0.68	-0.01	0.05
Genus (Eclectus)	0.98	0.45	1.50	< 0.001
Genus (Pionites)	-0.40	-0.94	0.15	0.16
Genus (Poicephalus)	-0.42	-0.93	0.08	0.10
Genus (Psittacus)	0.16	-0.03	0.36	0.10
BCS (1)	-0.90	-1.56	-0.24	0.01
BCS (2)	-0.07	-0.52	0.39	0.77
BCS (4)	0.45	0.13	0.77	0.01
BCS (5)	0.45	-0.05	0.95	0.08
Age	0.00	-0.01	0.01	0.80
Gender (female)	-0.12	-0.28	0.05	0.16
Diet (2)	-0.14	-0.39	0.11	0.28
Diet (3)	-0.11	-0.31	0.09	0.27
Reproduction (2)	-0.06	-0.24	0.12	0.51
Reproduction (3)	0.04	-0.17	0.25	0.72
Reproduction (4)	-0.68	-1.40	0.04	0.06
