# Supplementary material for: Serum Lipid Reference Intervals of High-Density, Low-Density and Non-High-Density Lipoprotein Cholesterols and Their Association with Atherosclerosis and Other Factors in Psittaciformes
Source: Animals (Basel). 2025 Aug 25;15(17):2493. doi: 10.3390/ani15172493 (PMC12427453; doi:10.3390/ani15172493)
Supplement: Supplementary file 1 [file animals-15-02493-s001.zip › animals-3796595-supplementary/Table S3.pdf]

**Predictors**	β	**CI (2.5%)**	**CI (97.5%)**	**p**
(Intercept)	3.39	2.93	3.85	< 0.001
Atherosclerosis (mild)	-0.18	-0.57	0.21	0.37
Atherosclerosis (moderate to heavy)	0.74	0.15	1.33	0.01
Genus (Ara and Anodorhynchus)	-1.27	-1.72	-0.82	< 0.001
Genus (Cacatua)	-0.36	-0.93	0.20	0.21
Genus (Eclectus)	1.02	0.14	1.90	0.02
Genus (Pionites)	-1.18	-2.09	-0.26	0.01
Genus (Poicephalus)	-0.67	-1.52	0.17	0.12
Genus (Psittacus)	0.15	-0.17	0.47	0.36
BCS (1)	1.42	0.34	2.50	0.01
BCS (2)	-0.37	-1.11	0.38	0.34
BCS (4)	0.46	-0.07	0.99	0.09
BCS (5)	0.29	-0.54	1.11	0.49
Age	0.00	-0.02	0.02	0.89
Gender (female)	-0.14	-0.41	0.13	0.30
Diet (2)	-0.30	-0.72	0.11	0.16
Diet (3)	-0.45	-0.77	-0.12	0.01
Reproduction (2)	0.10	-0.20	0.40	0.51
Reproduction (3)	0.22	-0.13	0.57	0.22
Reproduction (4)	-1.69	-2.87	-0.50	0.01
