# Supplementary material for: Serum Lipid Reference Intervals of High-Density, Low-Density and Non-High-Density Lipoprotein Cholesterols and Their Association with Atherosclerosis and Other Factors in Psittaciformes
Source: Animals (Basel). 2025 Aug 25;15(17):2493. doi: 10.3390/ani15172493 (PMC12427453; doi:10.3390/ani15172493)
Supplement: Supplementary file 1 [file animals-15-02493-s001.zip › animals-3796595-supplementary/Table S4.pdf]

**Predictors**	β	**CI (2.5%)**	**CI (97.5%)**	**p**
(Intercept)	2.89	2.33	3.46	< 0.001
Atherosclerosis (mild)	-0.17	-0.66	0.31	0.48
Atherosclerosis (moderate to heavy)	0.67	-0.06	1.40	0.07
Genus (Ara and Anodorhynchus)	-1.61	-2.17	-1.05	< 0.001
Genus (Cacatua)	-0.82	-1.52	-0.12	0.02
Genus (Eclectus)	1.91	0.81	3.01	0.001
Genus (Pionites)	-1.41	-2.55	-0.27	0.02
Genus (Poicephalus)	-1.05	-2.10	0.01	0.05
Genus (Psittacus)	-0.19	-0.59	0.22	0.36
BCS (1)	2.22	0.88	3.55	0.001
BCS (2)	-0.28	-1.20	0.64	0.55
BCS (4)	1.23	0.57	1.88	< 0.001
BCS (5)	1.54	0.52	2.56	0.001
Age	0.01	-0.01	0.03	0.44
Gender (female)	0.01	-0.33	0.34	0.98
Diet (2)	-0.31	-0.83	0.20	0.23
Diet (3)	-0.22	-0.63	0.18	0.29
Reproduction (2)	0.24	-0.13	0.62	0.20
Reproduction (3)	0.43	0.00	0.87	0.05
Reproduction (4)	3.61	2.14	5.08	< 0.001
