# Supplementary material for: Serum Lipid Reference Intervals of High-Density, Low-Density and Non-High-Density Lipoprotein Cholesterols and Their Association with Atherosclerosis and Other Factors in Psittaciformes
Source: Animals (Basel). 2025 Aug 25;15(17):2493. doi: 10.3390/ani15172493 (PMC12427453; doi:10.3390/ani15172493)
Supplement: Supplementary file 1 [file animals-15-02493-s001.zip › animals-3796595-supplementary/Table S5.pdf]

**Predictors**	**Log-Odds**	**CI (2.5%)**	**CI (97.5%)**	**p**
(Intercept)	-3.06	-4.71	-1.42	< 0.001
LDL-C	-0.19	-0.44	0.06	0.15
HDL-C	0.18	-0.11	0.47	0.21
Non-HDL-C	0.08	-0.10	0.31	0.43
Genus (Ara and Anodorhynchus)	0.39	-0.67	1.46	0.46
Genus (Cacatua)	0.88	-0.22	1.98	0.11
Genus (Eclectus)	-0.09	-2.07	1.89	0.93
Genus (Poicephalus)	0.00	-1.71	1.72	1.00
Genus (Psittacus)	1.18	0.50	1.85	0.001
BCS (1)	0.53	-2.96	4.01	0.67
BCS (2)	0.45	-1.38	2.28	0.62
BCS (4)	0.20	-0.86	1.27	0.74
BCS (5)	1.74	-0.04	3.52	0.09
Age	0.16	0.12	0.19	< 0.001
Gender (female)	-0.24	-0.83	0.36	0.42
Diet (2)	-1.17	-1.97	-0.37	0.004
Diet (3)	-1.71	-2.34	-1.08	< 0.001
Breeding (2)	0.84	-0.01	1.69	0.06
Breeding (3)	0.59	-0.87	2.05	0.43
