# Supplementary figures and images for: Serum Lipid Reference Intervals of High-Density, Low-Density and Non-High-Density Lipoprotein Cholesterols and Their Association with Atherosclerosis and Other Factors in Psittaciformes
Source: Animals (Basel). 2025 Aug 25;15(17):2493. doi: 10.3390/ani15172493 (PMC12427453; doi:10.3390/ani15172493)

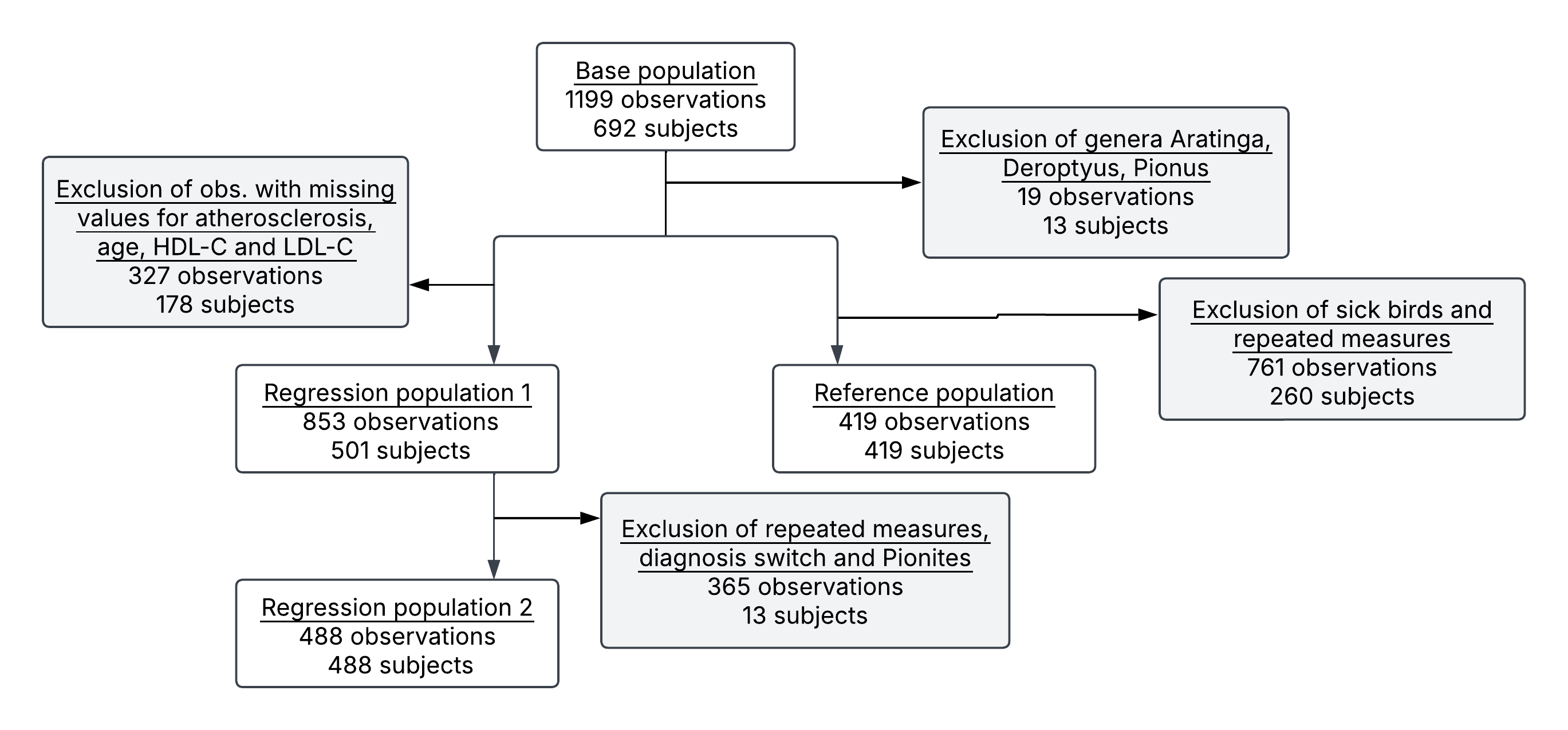

Supplement: Supplementary file 1 [file animals-15-02493-s001.zip › animals-3796595-supplementary/Figure S1.png]

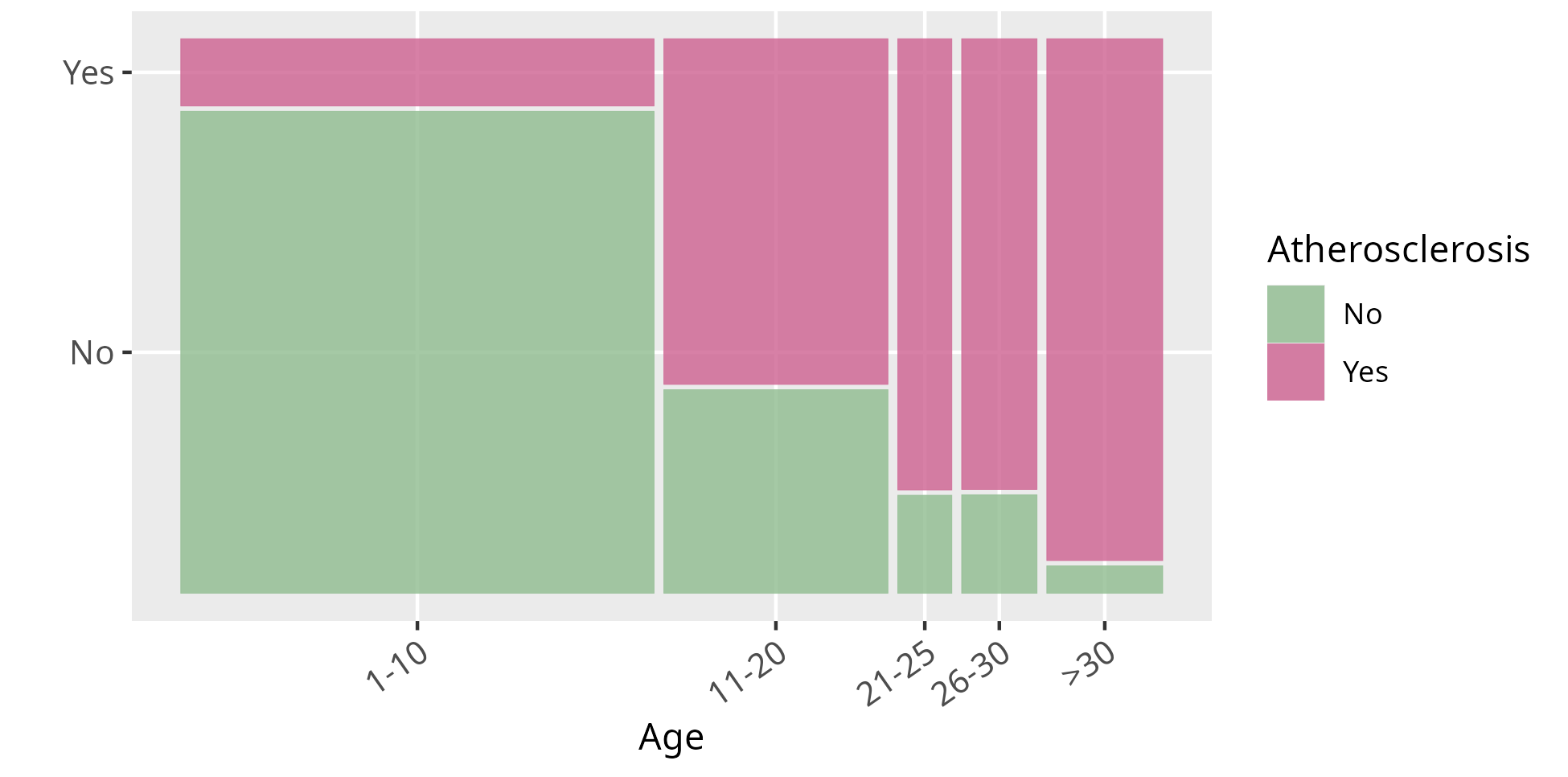

Supplement: Supplementary file 1 [file animals-15-02493-s001.zip › animals-3796595-supplementary/Figure S10.png]

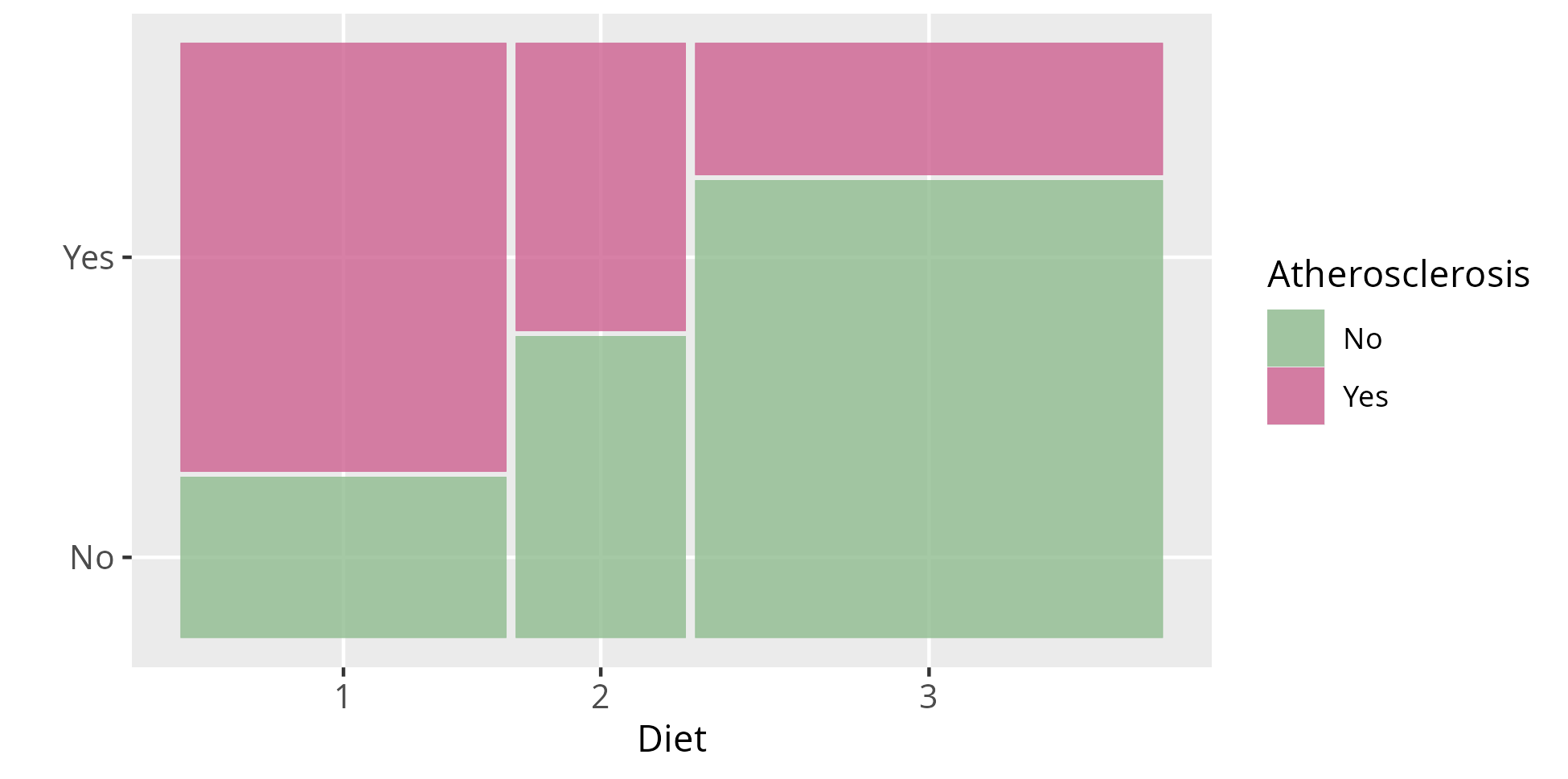

Supplement: Supplementary file 1 [file animals-15-02493-s001.zip › animals-3796595-supplementary/Figure S11.png]

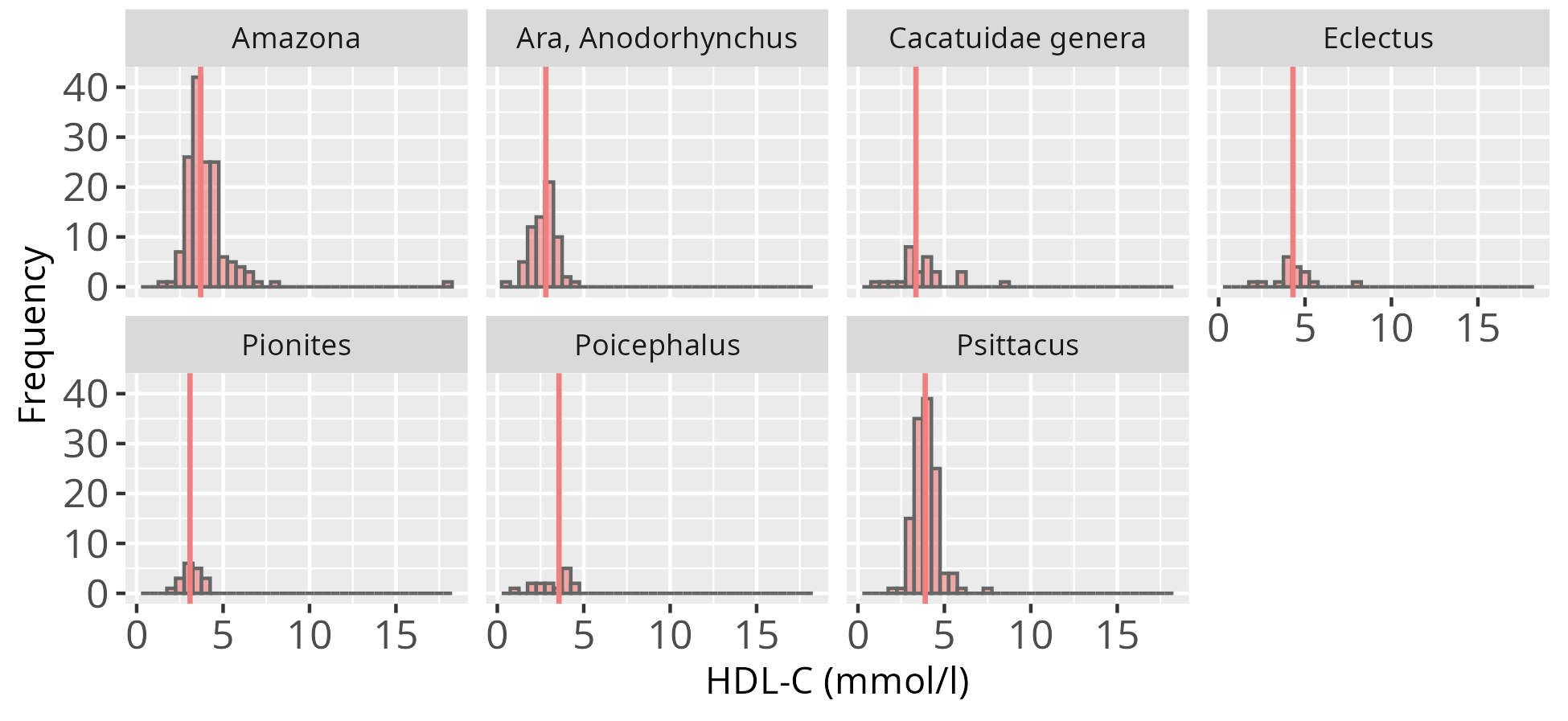

Supplement: Supplementary file 1 [file animals-15-02493-s001.zip › animals-3796595-supplementary/Figure S2.png]

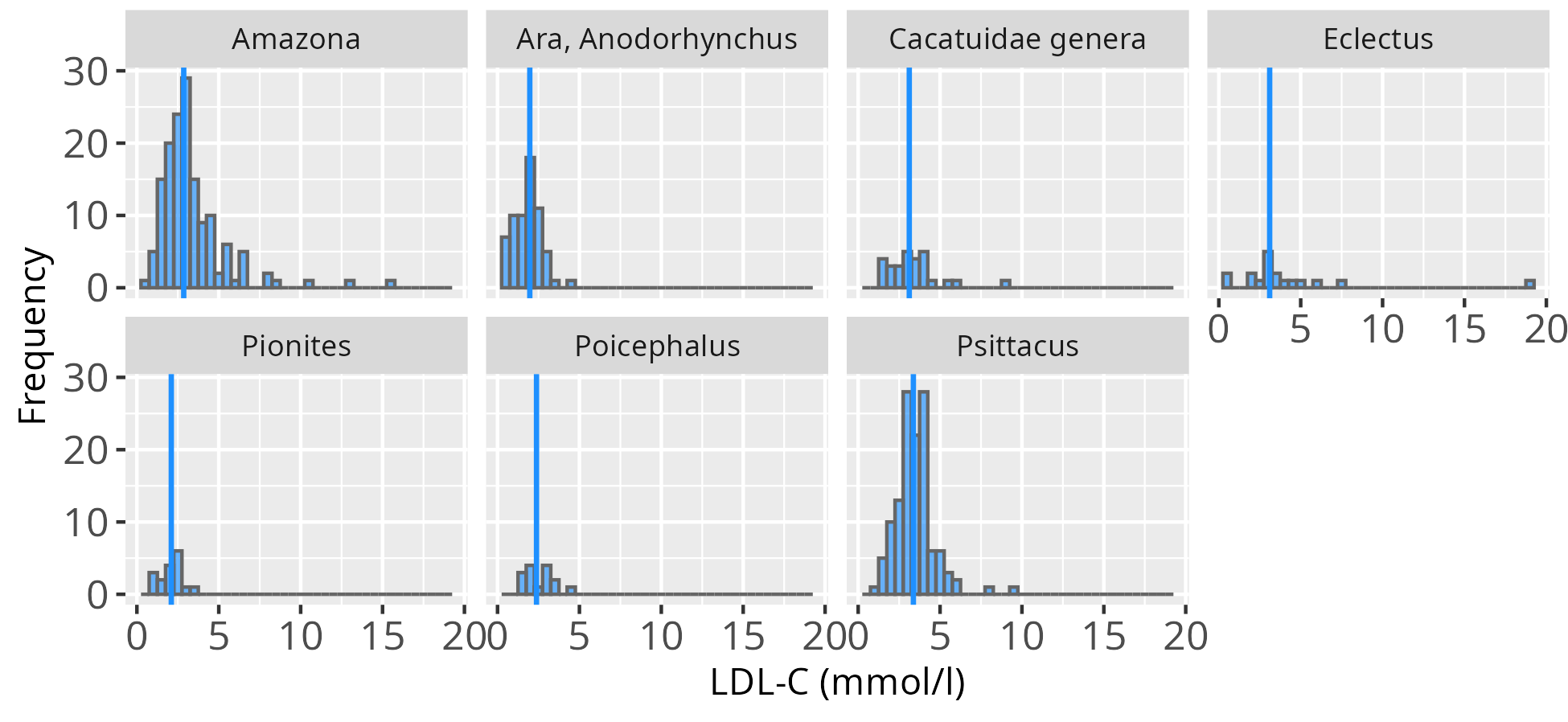

Supplement: Supplementary file 1 [file animals-15-02493-s001.zip › animals-3796595-supplementary/Figure S3.png]

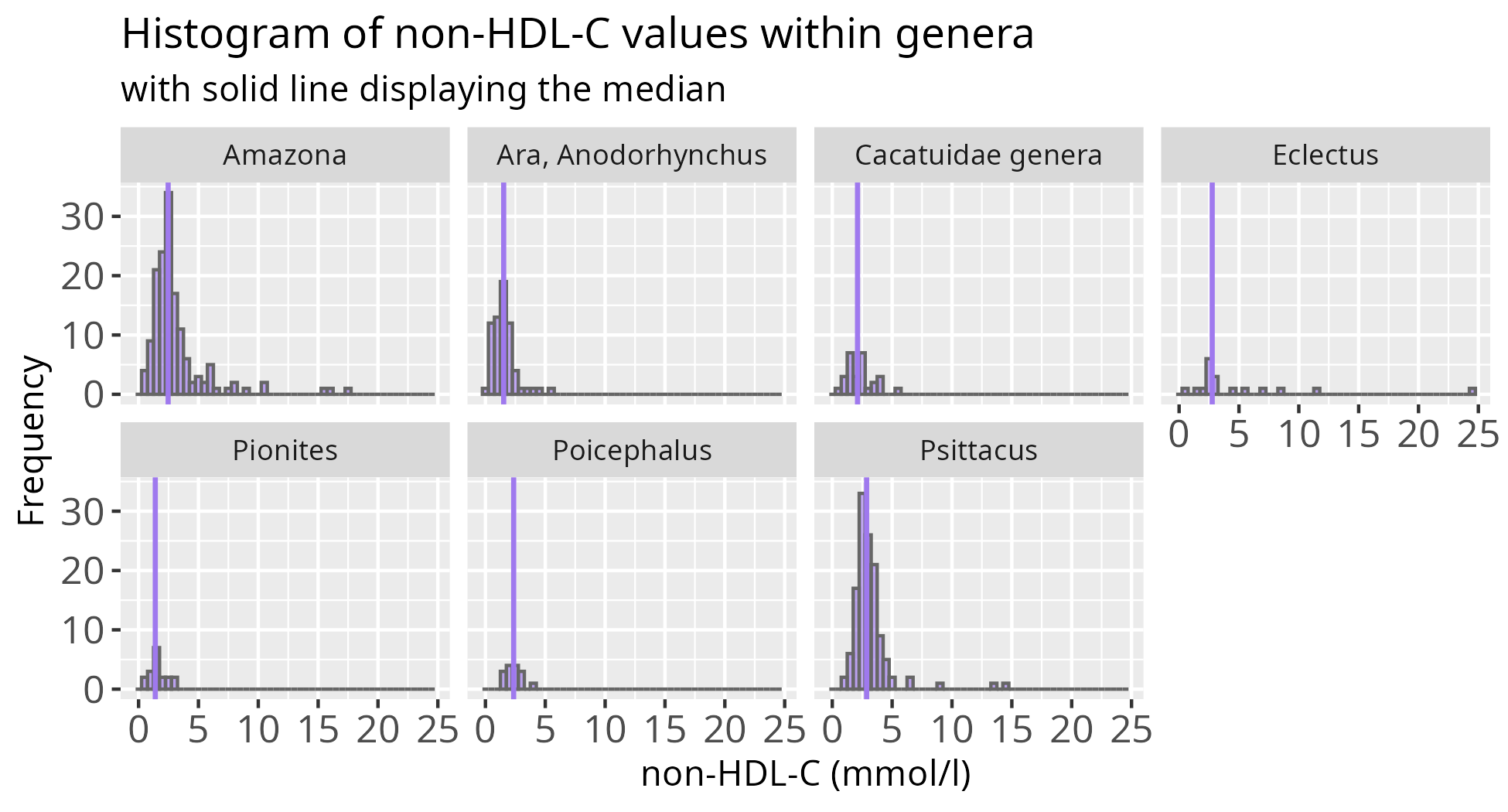

Supplement: Supplementary file 1 [file animals-15-02493-s001.zip › animals-3796595-supplementary/Figure S4.png]

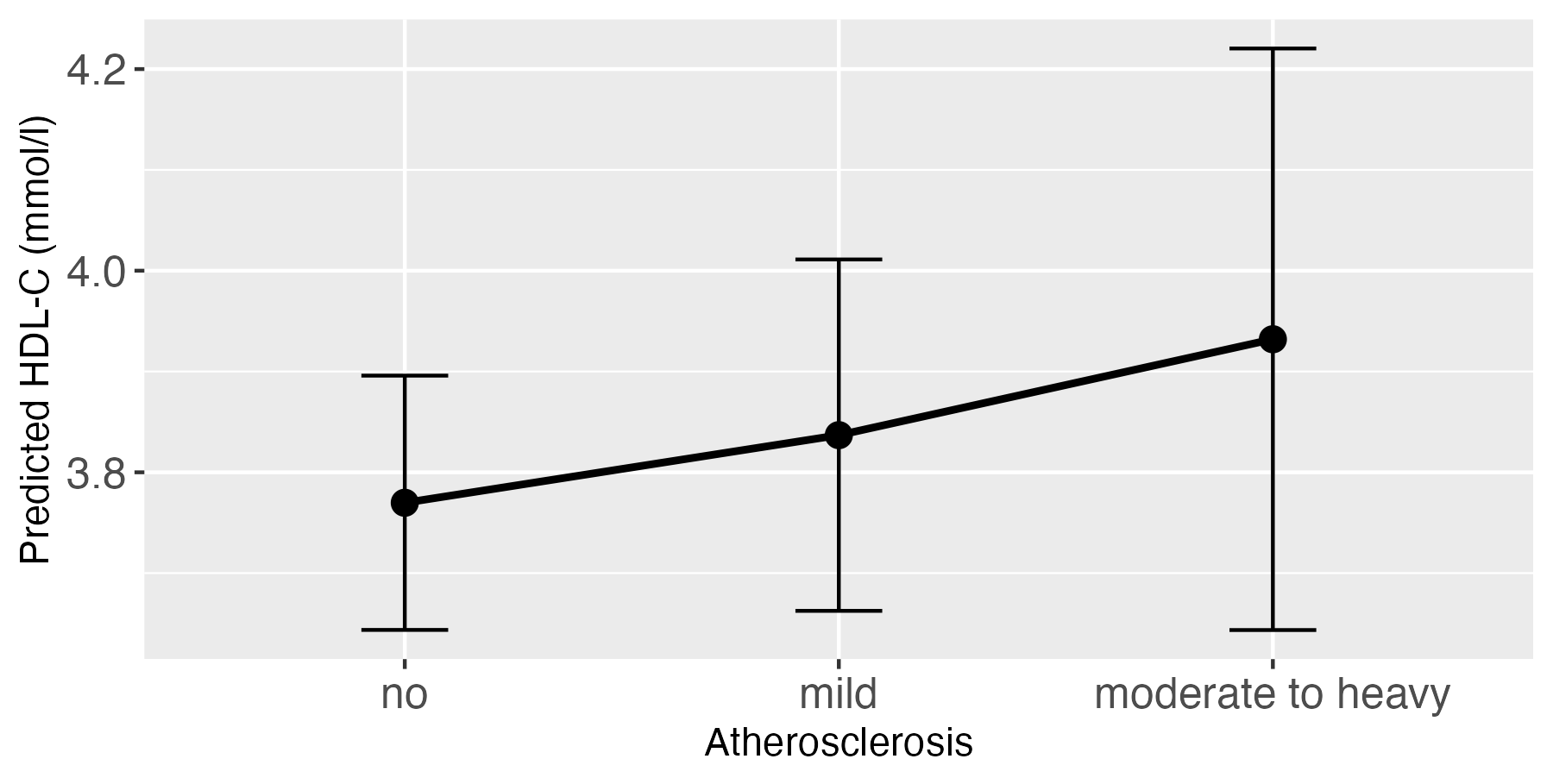

Supplement: Supplementary file 1 [file animals-15-02493-s001.zip › animals-3796595-supplementary/Figure S5.png]

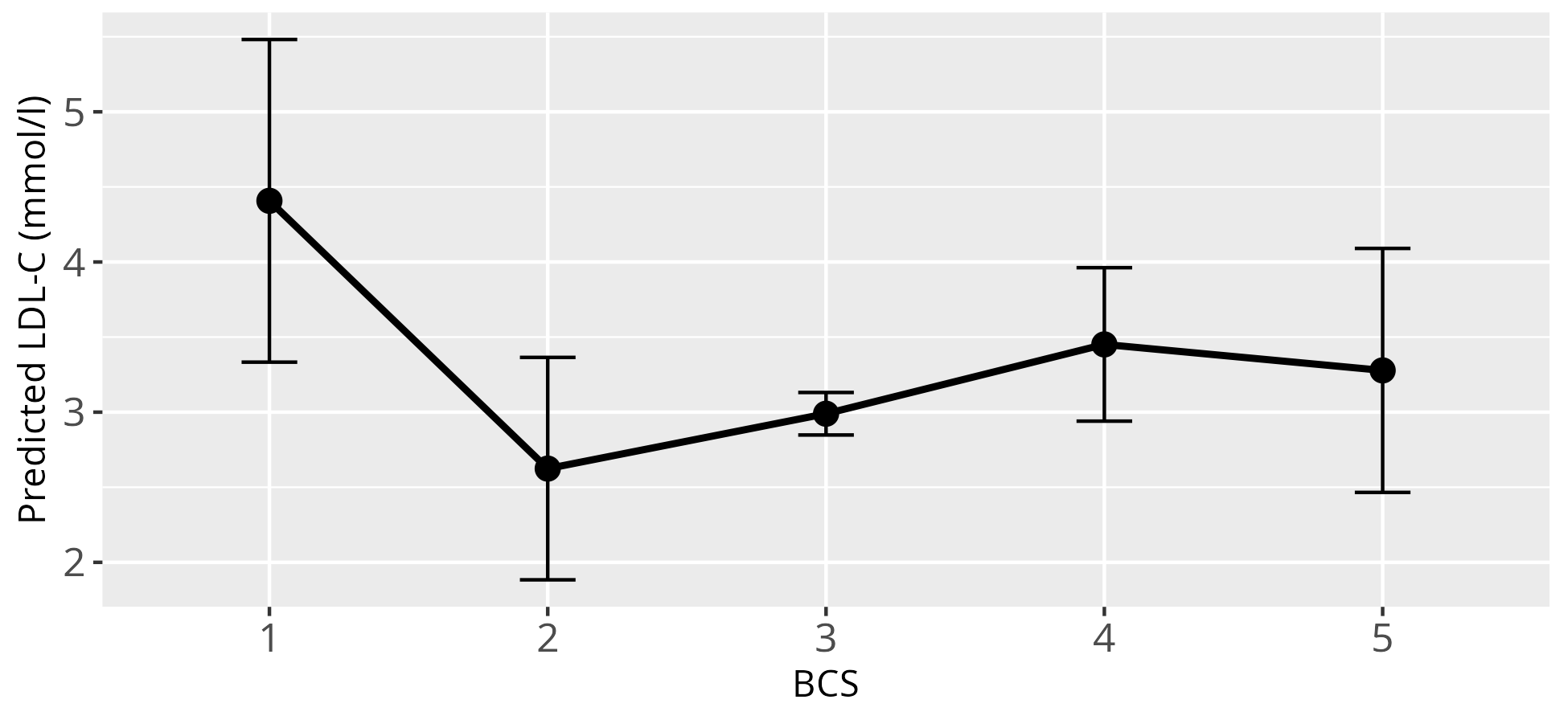

Supplement: Supplementary file 1 [file animals-15-02493-s001.zip › animals-3796595-supplementary/Figure S6.png]

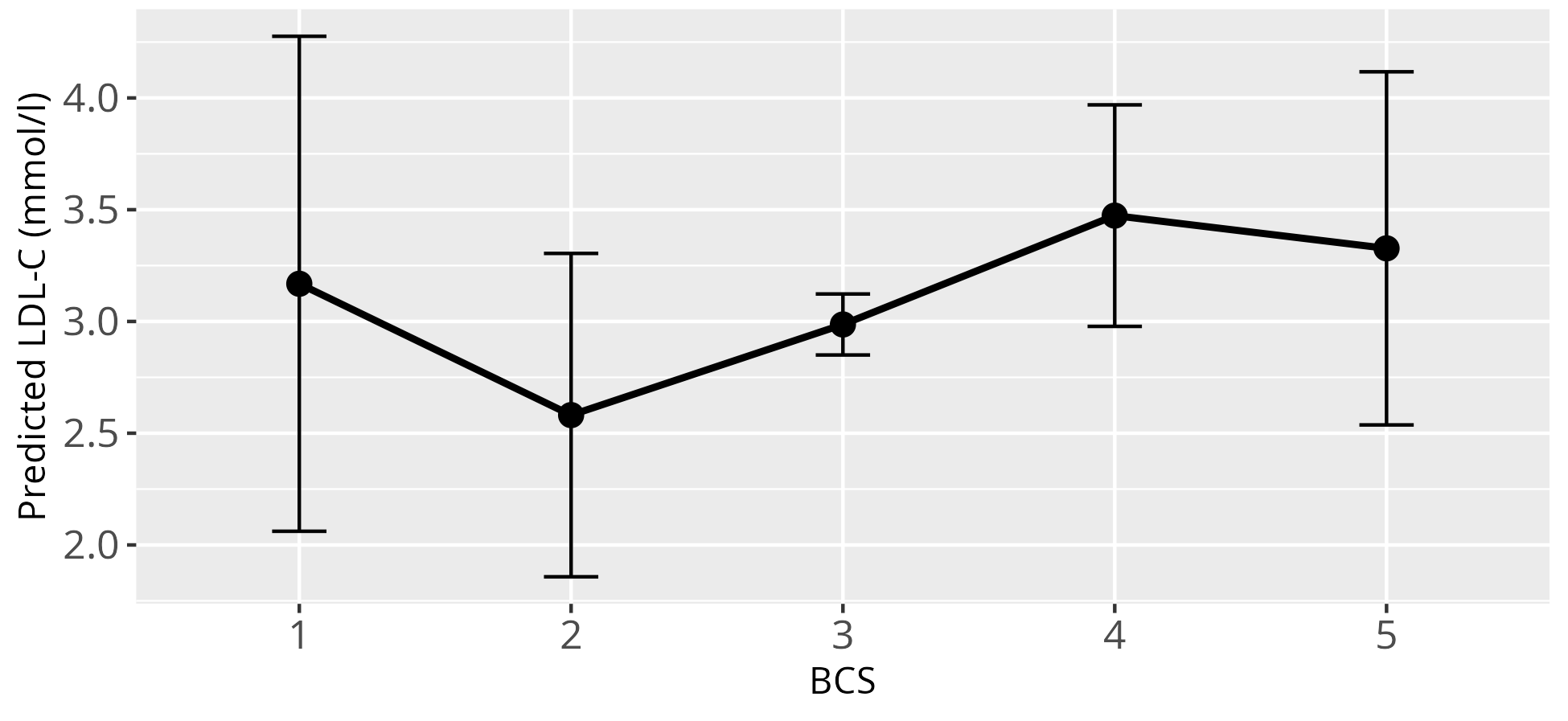

Supplement: Supplementary file 1 [file animals-15-02493-s001.zip › animals-3796595-supplementary/Figure S7.png]

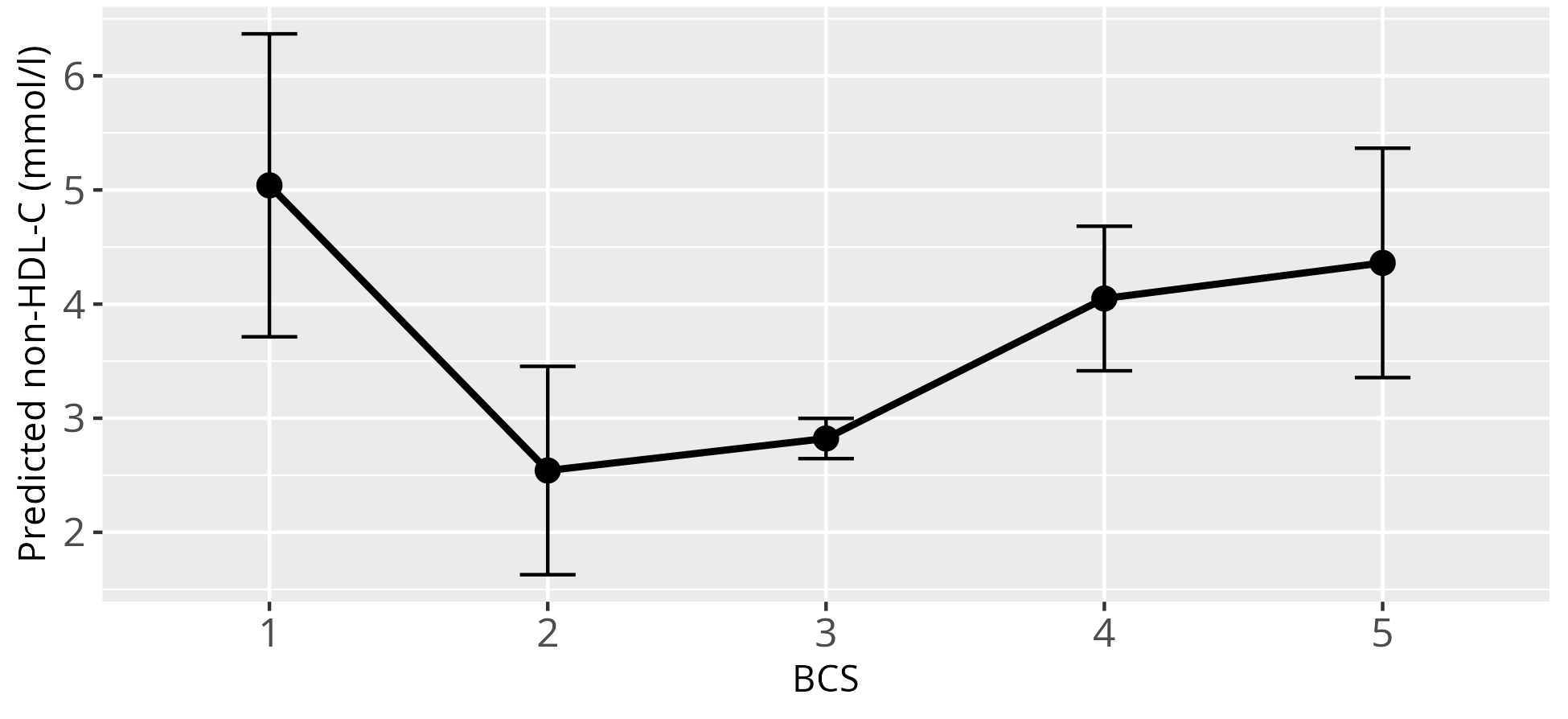

Supplement: Supplementary file 1 [file animals-15-02493-s001.zip › animals-3796595-supplementary/Figure S8.png]

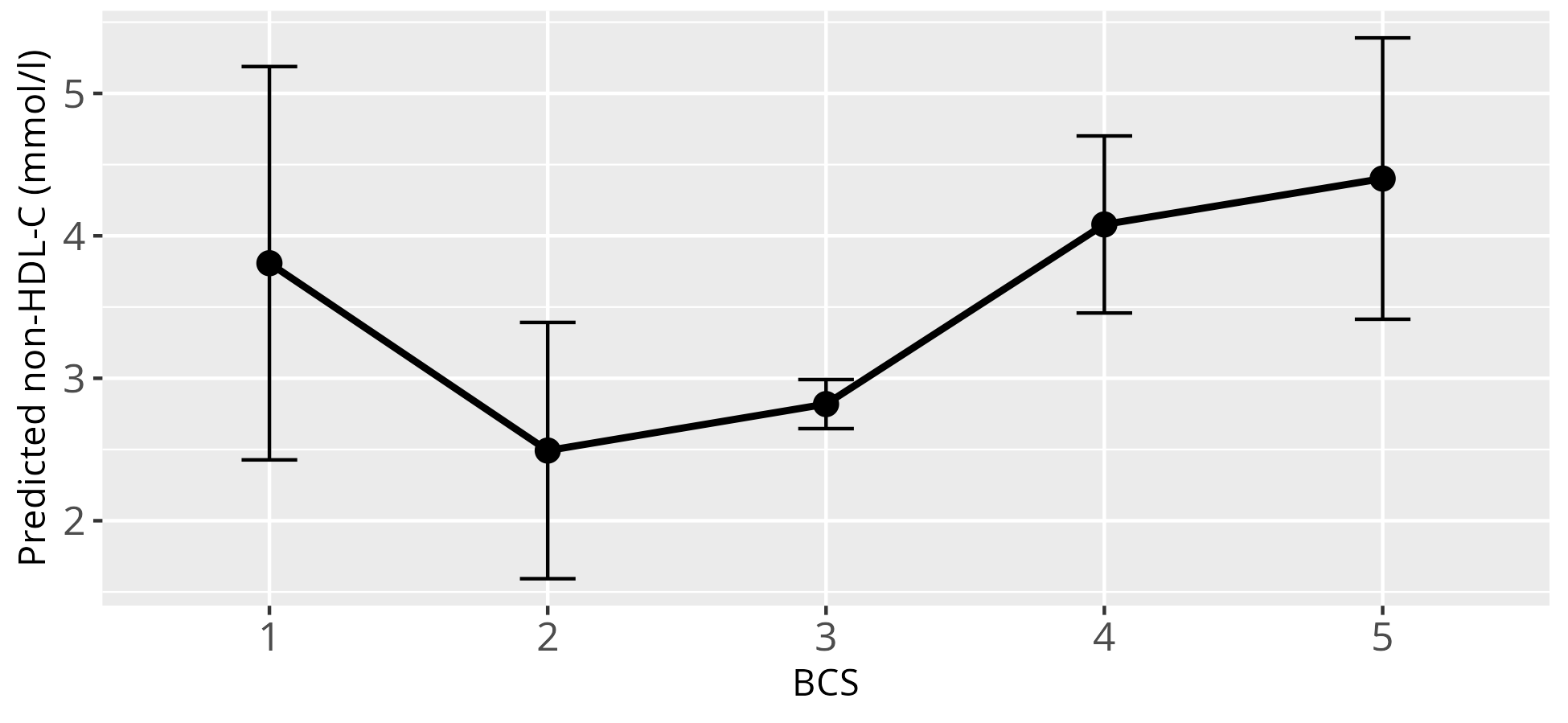

Supplement: Supplementary file 1 [file animals-15-02493-s001.zip › animals-3796595-supplementary/Figure S9.png]
